# Photocatalysis-mediated drug-free sustainable cancer therapy using nanocatalyst

**DOI:** 10.1038/s41467-021-21618-1

**Published:** 2021-03-01

**Authors:** Bin Zhao, Yingshuai Wang, Xianxian Yao, Danyang Chen, Mingjian Fan, Zhaokui Jin, Qianjun He

**Affiliations:** 1 Guangdong Provincial Key Laboratory of Biomedical Measurements and Ultrasound Imaging, National-Regional Key Technology Engineering Laboratory for Medical Ultrasound, Marshall Laboratory of Biomedical Engineering, School of Biomedical Engineering, Health Science Center, Shenzhen University, Shenzhen, Guangdong China; 2 Center of Hydrogen Science, Shanghai Jiao Tong University, Shanghai, China

**Keywords:** Combination drug therapy, Drug delivery

## Abstract

Drug therapy unavoidably brings toxic side effects and drug content-limited therapeutic efficacy although many nanocarriers have been developed to improve them to a certain extent. In this work, a concept of drug-free therapeutics is proposed and defined as a therapeutic methodology without the use of traditional toxic drugs, without the consumption of therapeutic agents during treatment but with the inexhaustible therapeutic capability to maximize the benefit of treatment, and a Z-scheme SnS_1.68_-WO_2.41_ nanocatalyst is developed to achieve near infrared (NIR)-photocatalytic generation of oxidative holes and hydrogen molecules for realizing combined hole/hydrogen therapy by the drug-free therapeutic strategy. Without the need of any drug and other therapeutic agent assistance, the nanocatalyst oxidizes/consumes intratumoral over-expressed glutathione (GSH) by holes and simultaneously generates hydrogen molecules in a lasting and controllable way under NIR irradiation. Mechanistically, generated hydrogen molecules and GSH consumption inhibit cancer cell energy and destroy intratumoral redox balance, respectively, to synergistically damage DNA and induce tumor cell apoptosis. High efficacy and biosafety of combined hole/hydrogen therapy of tumors are achieved by the nanocatalyst. The proposed catalysis-based drug-free therapeutic strategy breaks a pathway to realize high efficacy and low toxicity of cancer treatment.

## Abstract (web summary)

Nanoparticles have been used to reduce the toxicity associated with chemotherapeutic agents. Here, the authors report a Z-scheme SnS_1.68_-WO_2.41_ nanocatalyst for photocatalytic generation of oxidative holes and hydrogen molecules for drug-free therapeutic strategy.

## Introduction

A variety of nanomaterials are developed as drug carriers to encapsulate drug molecules and deliver them in a targeted way to the diseased site to enhance drug efficacy and reduce drug side effects^1–4^. As high drug loading capacity as possible is pursued all the way, but is always limited. Drug-free therapeutics, which is defined as a therapeutic methodology without the use of drug, without the consumption of therapeutic agents during treatment but with the inexhaustible therapeutic capability, will maximize the benefit of treatment and is thus worth expecting. Tang et al. developed a calcification method to kill cancer cells by injecting folic acid and Ca^2+^ into tumor, but these two therapeutic agents were consumed during cancer treatment^5^. Recently, Shi et al. has proposed the concept of nanocatalytic medicine to improve the efficacy of cancer therapy, which can realize drug-free therapy in principle in favor of evading toxic side effects of drug^6–10^. However, most of the developed catalytic nanomedicines have to load toxic drugs and easy-exhaustion therapy-assisted agents. The development of nanocatalytic nanomedicine for drug-free therapy is quite intriguing but still challenging. We here propose to develop drug-free nanocatalysts which can consume intratumoral endogenous substrates to generate therapeutic species for drug-free therapy of cancer.

Electrons and holes generated on the surface of catalyst have reductive and oxidative capabilities, respectively. In theory, electrons with enough high reductive capability (≥1.23 eV for H^+^-to-H_2_ evolution) can be used to reduce endogenous H^+^/CO_2_/NO_3_^‒^ into H_2_/CO/NO for gas therapy^11–13^, while holes with enough high oxidative capability (≥0.32 eV for GSH-to-GSSG evolution) can be utilized to oxidize glutathione (GSH) into GSSG to destroy the tumor microenvironment for cancer therapy. We here propose a concept of hole therapy, which is defined as a therapeutic methodology of using catalyst-generated holes for disease treatment. The development of wide energy band of catalysts possessing high oxidation and reduction capabilities for hole/gas therapy is vitally important, but still challenging.

**Fig. 1:**
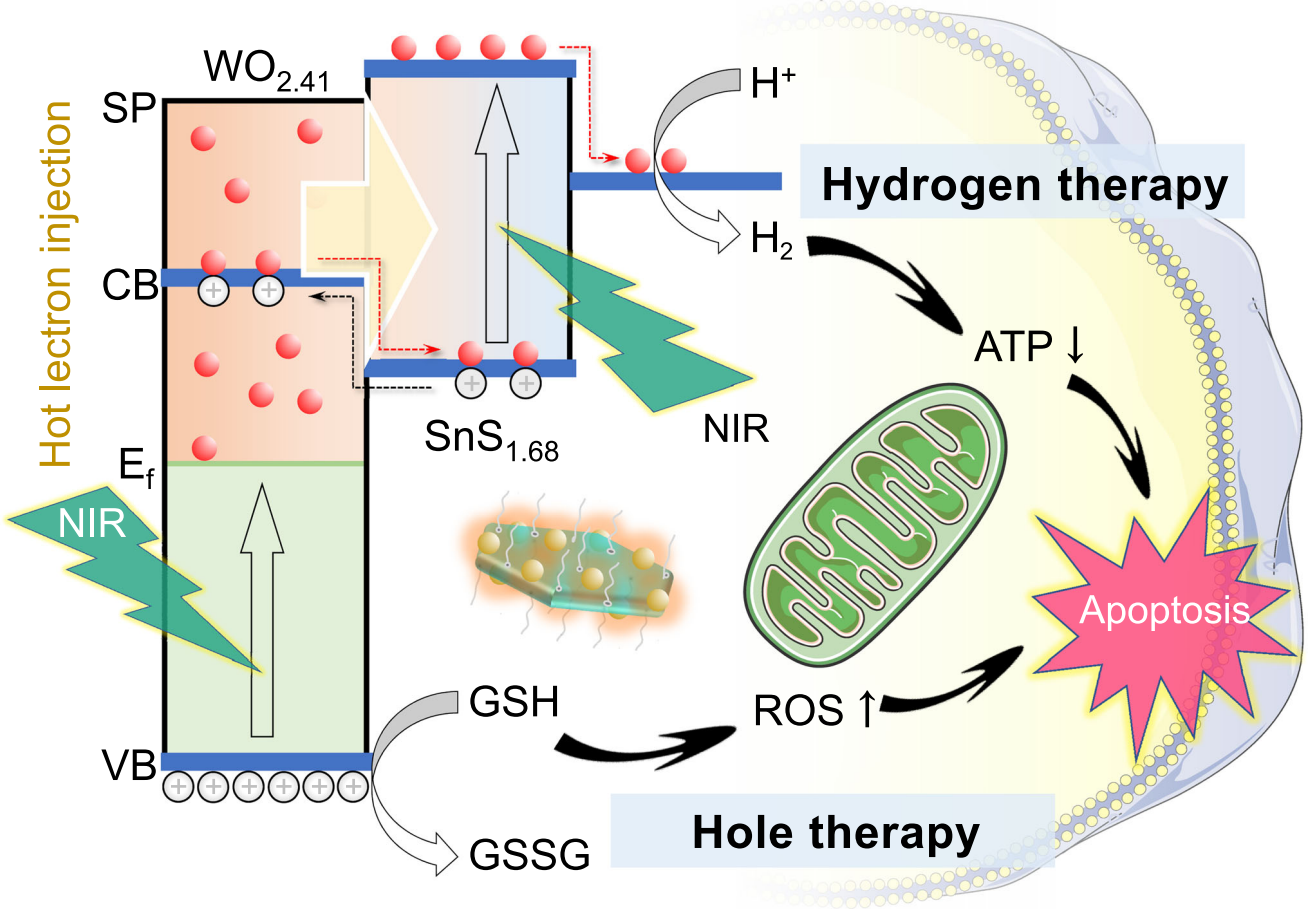
Schematic illustration of combined hole/hydrogen therapy strategy and mechanisms with the NIR-activable Z-scheme SnS_1.68_–WO_2.41_ nanocatalyst. Several main advances are achieved. (1) Advanced strategy of photocatalysis-mediated drug-free therapeutics is proposed. (2) Advanced Z-scheme SnS_1.68_–WO_2.41_ nanocatalyst is facilely constructed to achieve NIR-triggered efficient generation of highly oxidative holes and hydrogen molecules. (3) Advanced concept and mechanisms for combined hole/hydrogen therapy are proposed and uncovered. (4) Advanced therapeutic performances with drug-free and controllable generation of hydrogen and consumption of intratumoral over-expressed GSH are achieved by the nanocatalyst, realizing high biosafety and high efficacy of drug-free therapy.

The full utilization of NIR light energy at a high proportion (about 50%) in solar for photocatalytic hydrogen evolution is significant to hydrogen energy but also challenging at present, because NIR-photocatalysts possess narrow energy band width (≤1240/*λ*_NIR_, ≤1.53 eV with 808 nm NIR light) and too low oxidative capability (≥1.23 eV reduction potential of H^+^/H_2_)^14,15^. As to hydrogen medicine, NIR light as an external stimulus source for photocatalytic hydrogen evolution has obvious advantages over UV/visible light owing to its higher tissue penetrability and lower photo-toxicity^16^. However, the development of photocatalysts for NIR-driven hydrogen evolution for disease treatment is currently facing the similar issues to hydrogen energy. Recently, Sung et al. improved their photo-driven hydrogen-evolving liposome nanoreactor (citrate-UCNP-Au-Chlα@liposome) by encapsulating upconversion nanoparticle (UCNP) as NIR-to-visible transducer to realize NIR-photocatalytic production of hydrogen for anti-inflammation^17–19^. However, the content of encapsulated sacrificial agent citrate (6.0 wt%) is limited and the NIR-to-visible conversion efficiency of UCNP is poor, restricting the amount and efficiency of hydrogen evolution. Here, we hypothesize that the utilization of endogenous reductive substrates such as intratumoral over-expressed GSH (0.32 eV oxidation potential of GSH/GSSG) as sacrificial agent could realize long-lasting hydrogen production and drug-free therapy. Moreover, we propose to construct the Z-scheme structure with highly oxidative (low valence band) plasmatic nanodots and NIR-activable (narrow band gap) semiconductor to realize NIR-driven hot electrons injection and high-efficiency NIR-photocatalytic evolution of hydrogen molecules and GSH-oxidizing holes for combined hole/hydrogen therapy of cancer (Fig. 1).

As to the single-phase nanocrystal catalyst, NIR adsorption edge means narrow band gap (<1.53 eV for 808 nm light), slight oxidation at the same time of hydrogen generation, and easy recombination of separated electrons and holes. As to the Janus-like Z-scheme structure of nanocrystals such as MnS–Cu_7_S_4_ generally constructed by the ion-exchange approach, well-defined interfaces and well-exposed surfaces on both semiconductors favor efficient interfacial charge transfer and reduction/oxidation half-reactions^20^, but the candidate of catalytic materials is limited and the realization of NIR-photocatalytic hydrogen generation is challenging. In comparison, the Z-scheme structure could be constructed facilely by heterogeneous growth without limitation of the choice of catalytic materials^21^. In this work, the screening of two NIR-sensitive catalysts, SnS_1.68_ nanoplates with a high reductive potential and WO_2.41_ nanodots with high oxidative potential, for construction of the Z-scheme structure aimed for efficient hydrogen generation and GSH depletion for combined hydrogen/hole therapy. On the one hand, WO_2.41_ nanodots have an oxygen defect-induced localized surface plasmon resonance effect with strong NIR adsorption as well as a high oxidative potential in favor of GSH oxidation. On the other hand, SnS_1.68_ nanoplates possess a sulfur-deficient structure, leading to a narrow band gap (1.49 eV) and a NIR absorption edge (832 nm), in favor of NIR-photocatalytic hydrogen generation. The heterogeneous growth of WO_2.41_ nanodots on the surface of SnS_1.68_ nanoplates by a one-pot method enabled NIR-driven hot electrons injection into conjugated SnS_1.68_ nanoplates, enhancing NIR-photocatalytic reduction for hydrogen generation. Therefore, we chose the materials system of the Z-scheme SnS_1.68_–WO_2.41_ nanocatalyst to realize efficient NIR-driven hydrogen generation and GSH depletion.

In this work, we proposed a strategy of photocatalysis-mediated drug-free therapeutics, and developed a kind of Z-scheme NIR-photocatalyst by in situ conjugate WO_3-x_ nanodots onto SnS_2-y_ nanoplates. The NIR-excitated surface plasmatic resonance of WO_3-x_ injected hot electrons into SnS_2-y_ for NIR-controlled hydrogen evolution and synchronous GSH oxidation by holes (Supplementary Fig. 1). The simultaneous generation of hydrogen molecules and holes enabled hydrogen therapy and hole therapy, respectively, which inhibited cellular energy metabolism and destroyed cellular anti-oxidation defense system (ADS) by GSH decrease and subsequent ROS increase, synergistically inducing the apoptosis of cancer cells (Fig. 1).

## Results and discussion

**Fig. 2:**
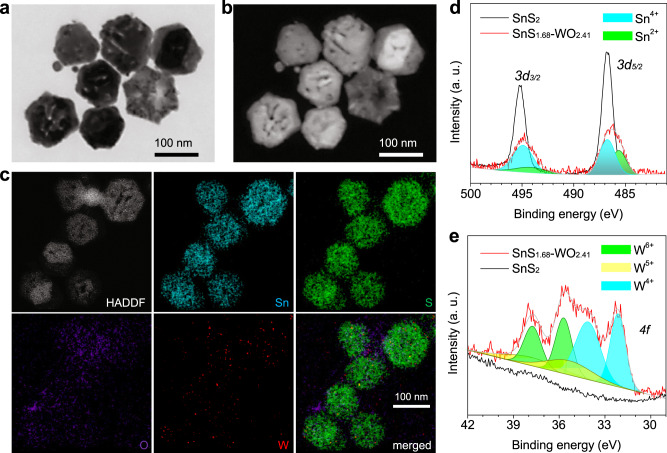
Morphology, composition, and size characterization of the SnS_1.68_–WO_2.41_ nanocatalyst. SEM (**a**) and corresponding STEM (**b**) images, HADDF image and corresponding elementary mapping (**c**), and XPS patterns (**d**, **e**) of the SnS_1.68_–WO_2.41_ nanocatalyst. The experiments were repeated three times independently with similar results.

SnS_2_ nanoplates with a stoichiometric proportion of Sn:S = 1:2 were prepared by a solvothermal method, and used as a control. WO_2.41_ nanodots heterogeneously grew on the surface of SnS_2_ nanoplates by the solvothermal method, and meanwhile, a part of sulfur was deprived by trimethylamine N-oxide (TMAO) during the oxidation of tungsten, leading to partial lack of sulfur and oxygen for SnS_1.68_–WO_2.41_. Through electrical microscopy observation, both obtained SnS_2_ and SnS_1.68_–WO_2.41_ exhibited a kind of hexagonal plate-like morphology (Fig. 2a‒c and Supplementary Fig. 2) as well as uniform size of about 100 nm in diameter in favor of cellular uptake. Elementary mapping and high-resolution TEM results suggested that there were small nanodots of tungsten oxide (at a 2.5% molar ratio of W to Sn, Supplementary Fig. 3) evenly dispersed on the surface of tin sulfide (Fig. 2c and Supplementary Fig. 2e) in favor of localized surface plasmon resonance (LSPR), which could be the reason that the surface of SnS_1.68_–WO_2.41_ nanoplates was rough and porous (Fig. 2a, b and Supplementary Fig. 2d). XPS patterns further indicated that the valence state of Sn in obtained SnS_2_ nanoplates was only 4+, while that in SnS_1.68_–WO_2.41_ nanoplates included 4+ and 2+ (Fig. 2d). The ratio of various Sn valence states in SnS_1.68_–WO_2.41_ was measured to be Sn^4+^:Sn^2+^ = 0.681:0.319, determining the non-stoichiometric ratio of Sn:S = 1:1.68. Such a sulfur-deficient structure of SnS_1.68_ led to the slight change in the crystal lattice of SnS_2_ (Supplementary Fig. 4b). Invisible XRD peaks of tungsten oxide in SnS_1.68_–WO_2.41_ nanoplates (Supplementary Fig. 4a) could result from its small particle size and low content. But FTIR results clearly indicated the composition of SnS_1.68_–WO_2.41_ (Supplementary Fig. 5) in accordance with the above elementary mapping results (Fig. 2c). XPS patterns further confirmed that there were three valence states of tungsten in SnS_1.68_–WO_2.41_ at the ratio of W^6+^:W^5+^:W^4+^ = 0.307:0.216:0.477 (Fig. 2e), determining the non-stoichiometric ratio of W:O = 1:2.41. So many oxygen vacancies in SnS_1.68_–WO_2.41_ nanoplates would greatly favor the enhancement of LSPR^22–24^. In addition, SnS_1.68_–WO_2.41_ nanoplates were conjugated with trithiol-terminated poly-(methacrylic acid) (PTMP-PMAA) by a coordination method to enhance their aqueous dispersion^25^. From DLS data in Supplementary Fig. 6, PTMP-PMAA-modified SnS_1.68_–WO_2.41_ nanoplates exhibited good dispersion in water and uniform particle size of about 150 nm in favor of passive tumor-targeted delivery. Additionally, the chemical stability of SnS_1.68_–WO_2.41_ nanoplates in the pH7.4 PBS was investigated. From Supplementary Fig. 7, after immersion in the PBS for 7 days, the morphology and adsorption property of SnS_1.68_-WO_2.41_ nanoplates were well maintained, and almost no free Sn and W ions can be detected by ICP, suggesting that SnS_1.68_–WO_2.41_ nanoplates were stable enough for therapy.

### Photoelectric behaviors and NIR-photocatalytic performances

**Fig. 3:**
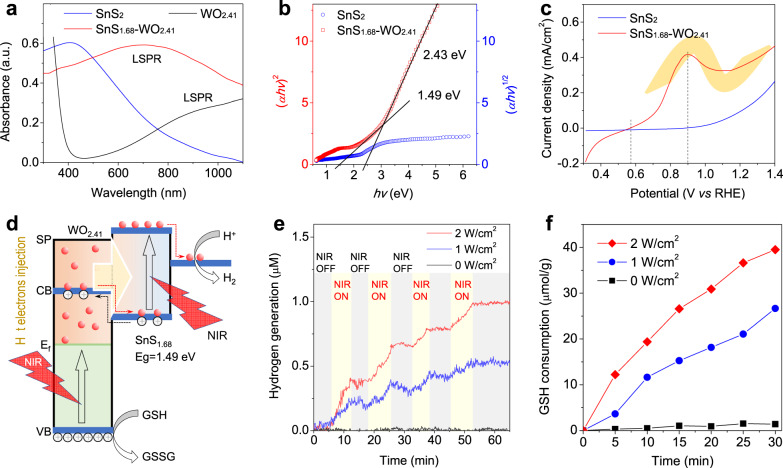
Photoelectric features and NIR-photocatalytic hydrogen generation and GSH consumption performances of the SnS_1.68_–WO_2.41_ nanocatalyst. UV–VIS–NIR adsorption spectra (**a**), the plots of (*αhν*)^1/2^ and (*αh*ν)^2^ versus energy (*hv*) for band gaps of SnS_2_ and SnS_1.68_–WO_2.41_ (**b**), photocurrent density–voltage (vs. RHE) curves of SnS_2_ and SnS_1.68_-WO_2.41_ under irradiation of 808 nm laser (**c**), the schematic illustration of the mechanism for NIR-photocatalytic hydrogen generation and GSH consumption (**d**), NIR-photocatalytic hydrogen generation behavior of the SnS_1.68_–WO_2.41_ nanocatalyst in the aqueous solution of GSH (10 μM) under irradiation of 808 nm laser at various power densities (**e**), NIR-photocatalytic GSH consumption behavior of the SnS_1.68_–WO_2.41_ nanocatalyst under irradiation of 808 nm laser at various power densities (**f**). The experiments for figures **a**, **e**, **f** was repeated three times independently with similar results.

From Fig. 3a, WO_2.41_ exhibited a strong LSPR absorption in the range of visible-to-infrared. In addition, SnS_2_ exhibited a broad UV‒visible‒NIR absorption and implied a narrow band gap. The mixture structure of SnS_1.68_–WO_2.41_ integrated NIR-absorption behaviors of WO_2.41_ and SnS_2_, creating necessary conditions for NIR-photocatalysis. Moreover, the oxygen-deficient structure of SnS_1.68_–WO_2.41_ could enhance LSPR, which will greatly favor the generation of hot electrons on its surface as expected^26–29^. From Fig. 3b, band gaps of SnS_1.68_ and WO_2.41_ within SnS_1.68_–WO_2.41_ were measured to be about 1.49 eV and 2.43 eV, respectively. So narrow band gap of SnS_1.68_ enabled NIR-photoelectric conversion at ≤832 nm, and can also provide an enough high reduction potential for H^+^/H_2_ evolution (1.23 eV). Furthermore, NIR-photocurrent measurement with 808 nm NIR laser indicated that the NIR-photocurrent onset potential (V_onset_) of SnS_1.68_–WO_2.41_ was as low as 0.57 V (Fig. 3c). By comparison, the NIR-photocurrent V_onset_ of SnS_2_ was much higher (0.9 V), where the NIR-photocurrent of SnS_1.68_–WO_2.41_ achieved the maximum (0.417 mA/cm^2^). It suggested that large amounts of hot electrons were easily yielded by WO_2.41_ on the surface of SnS_1.68_-WO_2.41_ under irradiation of 808 nm NIR laser owing to its LSPR effect, and then transferred and injected into SnS_1.68_ by a Z-scheme pathway, as demonstrated by the “Z” photocurrent curve in Fig. 3c. It could be found that both SnS_1.68_ and WO_2.41_ within the Z-scheme structure was photoelectrically sensitive to NIR light, greatly favoring the NIR-triggered generation and separation of electrons and holes^30,31^. According to the above-mentioned conclusion, we illustrated the Z-scheme photoelectric structure of the SnS_1.68_–WO_2.41_ nanocatalyst and its mechanism for NIR-photocatalytic hydrogen generation and GSH consumption in Fig. 3d. Additionally, more details for NIR-photocatalytic route were demonstrated in Supplementary Fig. 1. Under NIR excitation, holes (h^+^) and electrons (e^−^) were generated and separated on the surface of WO_2.41_ nanodots and SnS_1.68_ nanoplates, respectively. Holes on the surface of WO_2.41_ nanodots oxidized GSH into GSSG and generated H^+^ simultaneously. H^+^ transferred from WO_2.41_ nanodots to SnS_1.68_ nanoplates, and then was reduced by e^−^ into H_2_. In a word, GSH became into GSSG and H_2_ under the NIR-triggered photocatalysis of SnS_1.68_–WO_2.41_.

Furthermore, we checked the NIR-photocatalytic capability of the SnS_1.68_–WO_2.41_ nanocatalyst for hydrogen generation and GSH consumption. Noticeably, a hydrogen electrode was used to monitor the hydrogen generation process of the SnS_1.68_–WO_2.41_ nanocatalyst in real time, where catalyst-free detection solution was separated from NIR-illustrated nanocatalyst solution by a piece of filter membrane (molecular weight cut-off of 3000 Dalton) in order to avoid the effect of photoelectrons on the surface of nanoparticles on the electrode. From Fig. 3e, the SnS_1.68_–WO_2.41_ nanocatalyst can indeed generate hydrogen gas under irradiation of 808 nm laser, and the hydrogen generation rate depended on NIR laser power density, as higher power density caused faster hydrogen evolution. Moreover, hydrogen generation was highly controllable and repeatable by switching on/off NIR laser, which enabled on-demand hydrogen therapy. When NIR irradiation was switched off, hydrogen concentration went down a little bit owing to low solubility and density-induced hydrogen gas escape from the solution. Furthermore, GSH concentration was also monitored by an Ellmann probe (5,5′-dithiobis-(2-nitrobenzoic acid), DTNB). It was found from Fig. 3f that GSH consumption was dependent on NIR irradiation. No NIR irradiation almost did not cause GSH consumption, while higher NIR irradiation resulted in more GSH consumption, owing to GSH oxidation by photo-generated holes on the SnS_1.68_–WO_2.41_ nanocatalyst rather than by the nanocatalyst itself, enabling hole therapy. The GSH consumption seems to have a linear relationship with irradiation time, indicating the nanocatalyst can steadily oxidize GSH for generating hydrogen molecule with high cycle stability of NIR-photocatalysis. It is worth noting that such a drug-free therapy method is sustainable and repeatable without restrictions, empowering any extent of therapy for optimal outcomes.

### In vitro hole/hydrogen therapy efficacies and mechanisms

**Fig. 4:**
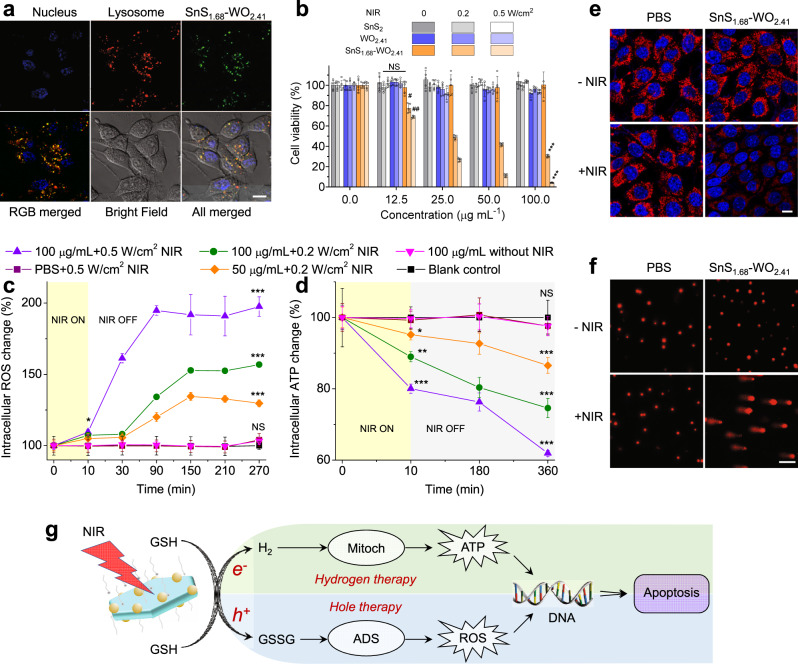
Combined hole/hydrogen therapy performances and mechanisms of the SnS_1.68_–WO_2.41_ nanocatalyst. The cellular uptake behavior of the SnS_1.68_–WO_2.41_ nanocatalyst after incubation for 4 h (**a**), the cytotoxicity (*n* = 5 biologically independent cells) of the nanocatalyst in the presence/absence of NIR irradiation (808 nm, 0.2 or 0.5 W/cm^2^, 10 min) against 4T1 cells (**b**), the intracellular ATP (**c**) and ROS (**d**) monitoring (*n* = 6 biologically independent samples) in SnS_1.68_–WO_2.41_-treated 4T1 cells in the presence/absence of NIR irradiation (808 nm, 0.2 or 0.5 W/cm^2^, 10 min), the effect of SnS_1.68_-WO_2.41_ + NIR treatment on the mitochondria (The experiments were repeated three times independently with similar results) (**e**) and DNA (**f**) of 4T1 cells, and the proposed mechanism for combined hole/hydrogen therapy based on the SnS_1.68_–WO_2.41_ nanocatalyst (**g**). *P* values were calculated by the two-tailed Student’s *t*-test (^#^*P* = 0.000253, ^##^*P* = 0.000007, *P = 0.001356, **P = 0.000006, ***P < 0.000001; NS, no significant difference). Scale bars, 10 μm (**a**, **e**), 100 μm (**f**). Data are presented as mean values ± SD.

Cellular uptake behavior of the SnS_1.68_–WO_2.41_ nanocatalyst was investigated first. From Fig. 4a, the SnS_1.68_–WO_2.41_ nanocatalysts were largely endocytosed through the lysosomal pathway by HeLa cells after 4 h incubation, owing to their small particle size in favor of cellular uptake. Effective cellular uptake of nanocatalysts would be favorable for cancer therapy. Furthermore, the cytotoxicity of nanocatalysts was investigated with SnS_2_ nanoplates and WO_2.41_ nanodots as two controls that cannot oxidize GSH and generate hydrogen molecules under NIR irradiation because of unqualified energy band structures. From Fig. 4b and Supplementary Fig. 8c, the control groups of SnS_2_ nanoplates and WO_2.41_ nanodots did not affect the viability of 4T1 and HeLa cells in the absence and presence of NIR irradiation. Even after incubation 48 h, no visible dark cytotoxicities of SnS_2_, WO_2.41_, and SnS_1.68_–WO_2.41_ within a wide particle concentration range of 37.5‒600 μg/mL were exhibited (Supplementary Fig. 8ab), indicating high cytocompatibility of nanocatalysts. By comparison, SnS_1.68_–WO_2.41_ nanoplates plus NIR irradiation exhibited obvious cytotoxicity to 4T1 and HeLa cells with IC50 = 32.6 μg/mL at 0.2 W/cm^2^, and IC50 = 17.1 μg/mL at 0.5 W/cm^2^ for 4T1 cells (IC50 = 52.3 μg/mL at 0.2 W/cm^2^, and IC50 = 20.1 μg/mL at 0.5 W/cm^2^ for HeLa cells) (Fig. 4b and Supplementary Fig. 8c), owing to the occurrence of NIR-photocatalysis. In order to check the effect of individual hole or hydrogen, ultraviolet (UV) light was used instead of NIR to irradiate SnS_2_ + 250 µM *L*-ascorbic acid (AA) or WO_2.41_ + 250 µM Na_2_S_4_O_6_ to generate only hydrogen molecules or holes, respectively. From Supplementary Fig. 9b, UV irradiation of SnS_2_ or WO_2.41_ can kill 4T1 cells mildly in a power density-/concentration-dependent way, suggesting that photo-generated holes and hydrogen molecules have anti-cancer effects, which are here defined as hole therapy and hydrogen therapy. By comparison, combined hole/hydrogen therapy groups of SnS_1.68_–WO_2.41_ plus UV irradiation demonstrated remarkably higher cytotoxicity at the same particle concentration and power density. Enhanced anti-cancer efficacies were attributed to the effective combination of hole therapy and hydrogen therapy, demonstrating the advantages of drug-free therapy in therapy efficacy.

It has well proved that the main target of hydrogen molecule is mitochondrion and thus hydrogen gas can induce cancer apoptosis by adjusting cellular energy metabolism through mitochondria^32,33^, while GSH depletion can do damage to the ADS of cancer because intratumoral over-expressed GSH has high reduction capability and play an essential role in maintaining intratumoral redox balance^34–38^. Therefore, we investigated the effect of the SnS_1.68_–WO_2.41_ nanocatalyst on both intracellular levels of ATP and ROS and the functions of mitochondria and DNA in the presence and absence of NIR irradiation in order to uncover the mechanism of hole/hydrogen therapy. First, catalytic GSH oxidation (Supplementary Fig. 10a) and hydrogen evolution (Supplementary Fig. 10b) were further confirmed in the model of 4T1 cells in accordance to the above-mentioned results in the simulated fluid (Fig. 3ef), which was responsible for above-mentioned hole/hydrogen therapy outcomes (Fig. 4b). Furthermore, intracellular ROS monitoring results from Fig. 4c indicated that 10 min NIR irradiation to SnS_1.68_–WO_2.41_ nanocatalyst-treated 4T1 cells quickly caused the visible increase of intracellular ROS level in a power density-/concentration-dependent way, but no NIR irradiation did not affect intracellular ROS level obviously. It is worth noting that intracellular ROS level persistently increased (up to two folds within 1.5 h at 0.5 W/cm^2^ at 100 μg/mL) after stopping NIR irradiation, indicating that the damage of GSH oxidation to the ADS can be gradually amplified and then persisted for a long term (>4.5 h, Fig. 4c). Such a remarkable and irreducible enhancement of intracellular ROS level led to visible DNA damage (Fig. 4f and Supplementary Fig. 11), being accountable for hole therapy. On the other hand, NIR-photocatalytically generated hydrogen gas severely impaired the mitochondria of 4T1 cells (Fig. 4e and Supplementary Fig. 12), and thus caused the remarkable reduction in cellular energy level (Fig. 4d). Moreover, the response of intracellular ATP to hydrogen molecules was synchronous with the response of intracellular ROS to GSH oxidation, and even more persistent in favor of sustained proapoptosis. The hydrogen-mediated decrease of cellular energy level can not only directly induce the apoptosis of cancer cells but also impede the repairing of DNA damage^38^, and therefore synergistically enhance the effect of hole therapy. By comparison, SnS_2_ nanoplates did not affect intracellular levels of ROS and ATP despite concentration and NIR irradiation (Supplementary Fig. 13), probably owing to their good cytocompatibility and impossible NIR-photocatalysis. Based on above conclusion, we proposed the mechanism of combined hole/hydrogen therapy based on the SnS_1.68_–WO_2.41_ nanocatalyst, as illustrated in Fig. 4g.

### In vivo hole/hydrogen therapy outcomes

**Fig. 5:**
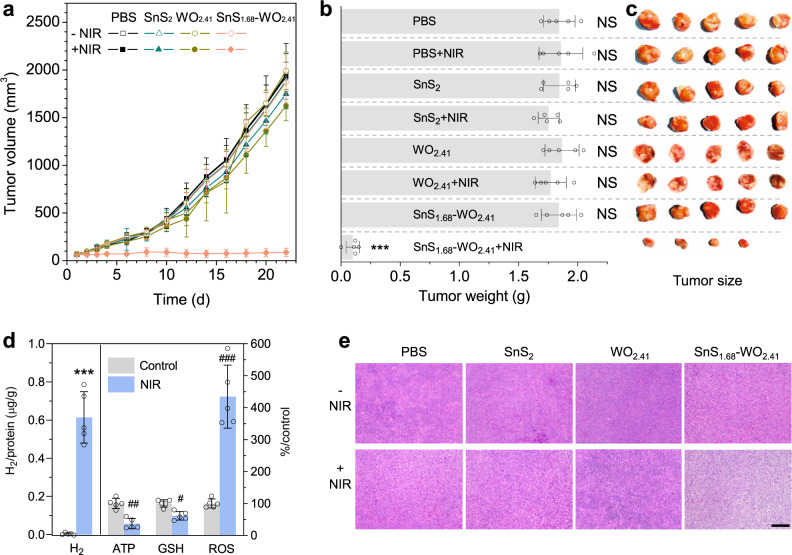
In vivo combined hole/hydrogen therapy performances of the SnS_1.68_–WO_2.41_ nanocatalyst. The monitoring of 4T1 tumor volume (**a**), the weight (**b**) and size (**c**) of extracted 4T1 tumors after 22-day treatment (*n* = 5 biologically independent samples), the intratumoral H_2_, ATP, GSH, and ROS levels (**d**) before and after NIR irradiation (20 min, 0.5 W/cm^2^) on nanocatalyst-treated tumor mice (*n* = 5 biologically independent samples), and the HE stained tumor tissue slices (**e**). Scale bar, 200 μm. *P* values were calculated by the two-tailed Student’s *t*-test (^#^*P* = 0.000653, ^##^*P* = 0.00007, ^###^*P* = 0.000015, ***P < 0.000001; NS, no significant difference). Data are presented as mean values ± SD.

In vivo tumor-targeted hole/hydrogen therapy efficacies of the SnS_1.68_–WO_2.41_ nanocatalyst were further investigated using two kinds of tumor mouse models built with murine 4T1 breast cancer cells and human HeLa cervical cancer cells. First, from ICP measurement results (Supplementary Fig. 14), the SnS_1.68_–WO_2.41_ nanocatalyst can effectively accumulate in the tumor site with a high accumulation efficiency (about 12%) and a relative long blood circulation time (about 1 h) after intravenous injection in a passive targeting way, owing to small and uniform particle size of nanocatalyst. During the 20 min NIR irradiation treatment, the temperature of irradiated tumors was monitored in real time by an infrared thermal imaging instrument and was found to always maintain below 40 °C, indicating that photothermal effect of the nanocatalyst was quietly slight. NIR irradiation was operated at day 1 and day 3. After treatment for 22 days, both 4T1 and HeLa tumors were effectively inhibited (Fig. 5a, Supplementary Fig. 15a), and even one of five 4T1 tumors was completely eradicated (Fig. 5b). By comparison, neither the SnS_1.68_–WO_2.41_ nanocatalyst without NIR irradiation nor the SnS_2_ nanoplates with/without NIR irradiation remarkably affected tumor growth of 4T1 tumors (Fig. 5b, c) and HeLa tumors (Supplementary Fig. 15b, c), owing to no generation of hydrogen and hole in these groups.

The proposed-above mechanism for combined hole/hydrogen therapy based on cell experiments (Fig. 4g) was further verified in vivo. After 0.5 W/cm^2^ NIR irradiation on tumors for 20 min, intratumoral GSH level sharply declined and hydrogen gas was generated largely (Fig. 5d), indicating intratumoral efficient NIR-photocatalytic hydrogen generation and GSH oxidation by SnS_1.68_–WO_2.41_ in good accordance with in vitro results. Meanwhile, intratumoral ROS and ATP levels intensively increased and decreased (Fig. 5d), respectively, in good consistence with in vitro results. Finally, most of the tumor cells treated with SnS_1.68_–WO_2.41_ plus NIR irradiation died without nuclei (blue, Fig. 5e), implying DNA damage. Therefore, in vivo results suggested that NIR-photocatalytical GSH oxidation caused the increase of intratumoral ROS level and thus damaged the DNA of tumor cells to induce tumor apoptosis, while NIR-photocatalytically generated hydrogen gas inhibited the energy level of tumor cells and consequently prevented from DNA repair to accelerate tumor apoptosis, further confirming the proposed mechanism for combined hole/hydrogen therapy (Fig. 4g).

**Fig. 6:**
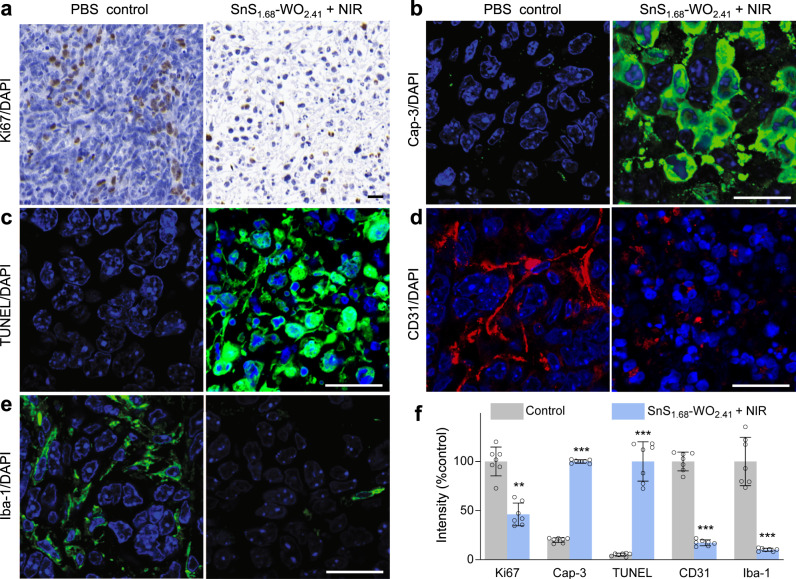
The change of TME after hole/hydrogen therapy with the SnS_1.68_–WO_2.41_ nanocatalyst. Immunohistochemical analysis of Ki67^+^ proliferating cells (**a**), caspase-3^+^ apoptotic cells (**b**), TUNEL^+^ apoptotic cells (**c**), CD31^+^ tumor vessels (**d**), and Iba1^+^ tumor-associated macrophages (**e**) in 4T1 tumors, and corresponding quantification results (*n* = 7 biologically independent samples) (**f**). All the scale bars, 20 μm. *P* values were calculated by the two-tailed Student’s *t*-test (**P = 0.000006, ***P < 0.000001). Data are presented as mean values ± SD.

Furthermore, we investigated the change of tumor microenvironment (TME) after hole/hydrogen therapy to deeply understand its influence. There are a variety of tumor-associated stromal cells, involving inflammatory cells, vascular cells, and fibroblasts, which make important contributions together to TME in support of tumor angiogenesis, proliferation, invasion, metastasis, and therapeutic resistance. Besides, tumor defense systems in TME safeguard the normal running of tumor system from internal and external attacks. Doing damage to TME by destroying tumor defense systems is equally important with directly killing tumor cells, and it has been well recognized that combined therapy for simultaneously damaging TME and killing tumor cells is more efficient than individual therapy, typically immunochemotherapy^39^. After hole/hydrogen therapy, the proliferation of Ki67^+^ tumor cells was almost thoroughly suppressed (Fig. 6a); both Caspase-3-positive (Cap3^+^) and terminal deoxynucleotidyl transferase-mediated deoxyuridine triphosphate nick end labeling-positive (TUNEL^+^) cellular apoptosis increased remarkably (Fig. 6b, c); excessive CD31^+^ vasculatures regressed drastically (Fig. 6d); and tumor-associated macrophages (Iba1^+^) were also almost fully eradicated (Fig. 6e), indicating that tumor-induced immunodepression was demilitarized. It could be found that the whole TME was completely altered by hole/hydrogen therapy, which was possibly one of main reasons of good therapy outcomes. By comparison, other control groups did not cause remarkable changes of TME (Supplementary Fig. 16). By virtue of hole/hydrogen therapy, both GSH depletion and hydrogen regulation upon tumor tissue could spread over the whole of TME, destroy the ADS in TME, and influence all tumor and tumor-associated stromal cells because of high tissue penetration of GSH and hydrogen molecules. Such a special feature of hole/hydrogen therapy is distinctly different from that of general drug therapies because of no drug resistance and no limitation of tissue penetration of nanoparticles, implying underlying clinical values of drug-free therapy.

Moreover, the biosafety of the SnS_1.68_–WO_2.41_ nanocatalyst was elevated by measuring its tissue and blood compatibilities since it distributed in multiple organs after intravenous injection. All treatment groups did not caused distinct body weight change (from Supplementary Figs. 17 to 18) or visible damage to main organs, including heart, liver, spleen, lung, and kidney after treatment for 22 days (from Supplementary Figs. 19 to  20), indicating good tissue compatibility of the SnS_1.68_–WO_2.41_ nanocatalyst. The general haematology parameters (red blood cells, white blood cells, haemoglobin, haematocrit, mean corpuscular haemoglobin, mean corpuscular haemoglobin concentration, and mean corpuscular volume) and standard blood biochemical indexes (alanine transaminase, aspartate transaminase, alkaline phosphatase, blood urea nitrogen, and creatinine) were assayed after intravenous injection of the SnS_1.68_–WO_2.41_ nanocatalyst (10 mg/kg) for 2 week. From Supplementary Fig. 21 to  22, there were no statistically significant differences in both haematology parameters and blood biochemical indexes between the SnS_1.68_–WO_2.41_ nanocatalyst and the control group, indicating good blood compatibility and no obvious toxicity to liver and kidney. It is noticeable that no NIR-irradiation did not trigger photocatalytic reactions and consequently did not cause toxicity to normal organs, and there was no toxic side effects of drug because no drug was used, reflecting the incomparable advantages of drug-free therapy in biosafety. Additionally, the toxicity of tungsten as a heavy metal is mainly demonstrated by soluble tungstates rather than by relatively more stable tungsten oxide and metal tungsten^40–48^, and therefore the development of tungsten oxide for biomedical applications is broadly being studies^42–48^ where high biocompatibility with lowest toxic dose of 1000 mg/kg for oral administration was demonstrated. In this work, we also found that neither WO_3_ nor SnS_1.68_–WO_2.41_ exhibited obvious cytotoxicity (600 μg/mL, Supplementary Fig. 8), side effect on intracellular GSH, mitochondria, and DNA (Supplementary Figs. 10–12), mice body weight change, tissue toxicity, and damage to liver/kidney functions (100 mg/kg for intravenous injection, Supplementary Figs. 17–22). For future clinical application, both local injection and nanocatalyst-encapsulating microneedle patch are optional administration routes for treatment of superficial tumors to avoid the risk of potential long-term toxicity of the nanocatalyst to normal tissues.

In summary, we developed the Z-scheme SnS_1.68_–WO_2.41_ nanocatalyst by the one-pot method to realize NIR-photocatalytic generation of holes and hydrogen molecules for combined hole/hydrogen therapy. Large amounts of hot electrons were easily yielded by plasmatic WO_2.41_ nanodots and injected into NIR-sensitive SnS_1.68_ nanoplate by a Z-scheme pathway, enabling efficient NIR-photocatalysis. The SnS_1.68_–WO_2.41_ nanocatalyst exhibited controlled NIR-photocatalytic hydrogen generation and GSH oxidation in vitro and in vivo, and effectively killed cancer cells and inhibited tumor growth by depressing cancer cell energy metabolism, enhancing intratumoral ROS level and inducing DNA damage. By the drug-free therapy strategy, the nanocatalyst harvested great benefits in the efficacy and biosafety of cancer therapy. Overall, the proposed catalyst-based drug-free therapy is a promising strategy for clinical cancer treatment.

## Methods

### Synthesis of SnS_2_ nanoplates

SnCl_4_·5H_2_O (0.3 mmol) and oleylamine (1.5 mL) was dissolved into anisole (28 mL) and then the mixture was slowly heated to 70 °C in oil bath. After stirred for 30 min, the solution became colorless and transparent, and then was cooled down to room temperature. CS_2_ (0.75 mL) was added under stirring. After 30 min, the solution turned into transparent yellowish color. The solution was heated to 180 °C in a polytetrafluoroethylene lined autoclave of 50 mL. After 12 h, the solution was cooled to room temperature, and then as-prepared SnS_2_ nanoplates were collected by centrifugation and washed twice with anhydrous ethanol^49^.

### Synthesis and modification of SnS_1.68_–WO_2.41_ nanoplates

The oleylamine solutions of SnS_2_ nanoplates (2 mL, 10 mg/mL) and W(CO)_6_ (1 mL, 5 mg/mL) were mixed with oleylamine (17 mL). After stirring at 60 °C for 30 min, the oleylamine solution of trimethylamine N-oxide dihydrate (TMAO, 1 mL, 4.75 mg/mL) was added into the above solution. After stirred for another 30 min, the solution was quickly heated to 260 °C under the protection of Ar atmosphere. After 12 min, toluene (10 mL) was dropwise added into the solution to quickly cool it to room temperature in order to quench the reaction. The as-prepared SnS_1.68_–WO_2.41_ nanoplates were collected by centrifugation, washed three times with the mixture of hexane (8 mL) and methanol (12 mL), and dispersed into ethanol for the following use. As a control, WO_2.41_ nanodots were synthesized by the similar route without the addition of SnS_2_ nanoplates.

The nanoplates were modified with trithiol-terminated poly-(methacrylic acid) (PTMP-PMAA) by the coordination route. In a typical procedure, the ethanol (100 mL) solution of methacrylic acid (MAA monomer, 10.888 g), pentaerythritol tetra(3-mercaptopropionate) (PTMP, 1.222 g) and azodiisobutyronitrile (AIBN, 0.2 g) was refluxed at 75 °C for 5.5 h under magnetic stirring and protection of Ar atmosphere. The solvent ethanol was removed by rotary distillation, and then the viscous product PTMP-PMAA was isolated by precipitation with cold anhydrous ether (4 °C), and then dried under vacuum at 45 °C for 24 h. The ethanol solution of PTMP-PMAA (1 mL, 10 mg/mL) was slowly dropped into that of nanoplates (5 mg, 10 mL) in an ultrasound bath. After sonication for 1 h, the solution was stirred for 24 h. The PTMP-PMAA modified nanoplates were collected by centrifugation and washed three times with ultrapure water to remove excessive PTMP-PMAA.

### Characterization of nanoplates

The morphology and size of nanoplates were measured by SEM (APREO, FEI) and TEM (JEM-2100F). Hydrodynamic size of nanoplates was measured on a Malvern Zetasizer Nano ZS90. The composition of nanoplates was determined by Fourier transform infrared spectroscopy (FTIR) on a Thermo-Nicolet Nexus 670 ATR-IR spectrometer. The UV absorption spectra were recorded on a Genesys 10S UV–Vis spectrophotometer. The photocurrent of nanoplates-coated indium tin oxide (ITO) glass was measured on a CHI 660D electrochemical station (Shanghai Chenhua, China) under irradiation of 1 W/cm^2^ 808 nm monochromatic light. During the test, a standard three-electrode device with the ITO glass as the working electrode, the platinum foil as the counter electrode, and the Ag/AgCl electrode as the reference electrode, was used. Three electrodes were inserted into the quartz battery pack and the photocurrent was measured in 0.5 M Na_2_SO_4_ electrolyte. All the data were saved as a txt file format and redrawn/analyzed in Origin 8.5 or GraphPad Prism 9, and figure panels were integrated in Microsoft Office PowerPoint 2007. The chemical stability of the nanocatalyst in the pH7.4 PBS was investigated by SEM, UV, and inductively coupled plasma-atomic emission spectrometry (ICP-AES, Agilent Technologies, USA). After immersion in PBS (30 μg/mL, 10 mL) for fixed time periods (0, 3, 7 days), the UV adsorption curve of the PBS solution of the nanocatalyst was measured, and nanoparticles were collected using the Millipore Ultra-Centrifugal Filter (molecular weight cut-off of 3000 Dalton) for TEM measurement, and the corresponding supernatant was used for ICP measurement (*n* = 3).

### Detection of hydrogen production in vitro and in vivo

Hydrogen concentration was quantitatively monitored with a hydrogen microelectrode (Unisense, Denmark). In the simulated solution, the reaction solution of nanoplates (100 μg/mL) and GSH (10 μM) was gently stirred under protection of nitrogen, and separated with the detection solution by a piece of filter membrane (molecular weight cut-off of 3000 Dalton). The light spot of 808 nm laser completely covered the reaction solution rather than hydrogen microelectrode in the detection reaction solution once the beginning of hydrogen detection. The controllability of NIR-triggered hydrogen production was measured by repeatedly switching on/off the laser at fixed time points in the dark.

In vitro, 4T1 cells were incubated with nanoplates (100 μg/mL) for 12 h, and then illustrated with 808 nm laser (0.5 W/cm^2^) for 10 min in the dark, and finally lysed by rapid freeze-thaw twice. Immediately, the supernatant was collected by centrifugation, and used to detect hydrogen concentration with hydrogen electrode. In vivo, 4T1 tumors of mice treated with nanoplates (10 mg/kg) for 4 h were illustrated with 808 nm laser (0.5 W/cm^2^) for 20 min in the dark, then extracted and quickly lysed under the assistance of grinding. Immediately, the supernatant was collected by centrifugation for detection of hydrogen concentration with hydrogen electrode. Meanwhile, intracellular and intratumoral protein contents were detected by the BCA kit (Beyotime Biotech.) for calculation of relative hydrogen levels.

### Detection of GSH depletion in vitro and in vivo

GSH concentration was quantitatively determined using the Ellmann probe (5,5′-dithiobis-(2-nitrobenzoic acid), DTNB). In the simulated solution, the natural light-sealed reaction solutions of nanoplates (100 μg/mL) and GSH (10 μM) were irradiated with different power densities (0, 1, 2 W/cm^2^) of 808 nm laser for different time periods (5‒30 min). The nanoparticles in the solution were removed by centrifugation, and the supernatant of 20 μL was added to the 10 mL 25 μM DTNB solution and then diluted to 3 mL with pH = 8.0 phosphate buffer solution for UV measurement. The decrease of the absorbance at 412 nm was collected to calculate the concentration and consumption of GSH using the linear standard curve of DTNB solutions according to the Beer–Lambert law. Similar to hydrogen detection, lysed solutions of 4T1 cells and tumors which were respectively illustrated with 808 nm laser (0.5 W/cm^2^) for 10 min and 20 min in the dark were collected for detection of in vitro and in vivo GSH depletion by the above Ellmann probe method.

### Detection of intracellular and intratumoral levels of ATP and ROS

The intracellular and intratumoral levels of ATP and ROS were detected by ATP and ROS assay kits (Beyotime Biotech.), respectively. Similar to hydrogen and GSH detection, 4T1 cells and tumors were illustrated with 808 nm laser (0.5 W/cm^2^) for 10 min and 20 min, respectively. After 3 h, 4T1 cells and tumors were lysed and the supernatant was immediately collected by centrifugation for detection of ATP and ROS levels in vitro and in vivo by corresponding assay kits.

### Measurement of cellular uptake of nanoplates

The nanoplates were labeled with FITC-PEG-SH by the coordination method similar to the PTMP-PMAA modification. 4T1 cells were seeded in a 6-well plate containing slides at the density of 1 × 10^5^ cells/well, and cultured with nanoplates at the final concentration of 100 μg/mL for 4 h. The lysosomes and nuclei of 4T1 cells were stained with Lyso-Tracker Red and Hoechst (Beyotime Biotech.), respectively. After removal of excessive dyes, 4T1 cells were observed on a confocal laser scanning microscope (ZEISS LSM880).

### Intracellular DNA and mitochondrial damage measurement

4T1 cells were cultured in a nanoplates-containing medium (100 μg/mL) for 12 h, and then irradiated with 0.5 W/cm^2^ 808 nm laser for 10 min. After incubation for another 3 h, 4T1 cells were stained with MitoTracker® Red CMXRos (Beyotime Biotech.). After incubation for another 20 min, excessive dyes were removed by washing with fresh medium, and then 4T1 cells were observed on a confocal laser scanning microscope (ZEISS LSM880) and meanwhile red fluorescence intensity per cell was calculated with ImageJ software to determine the intracellular mitochondrial amount. On the other hand, nanoplates-treated cells were digested by trypsin and washed with PBS, and then intracellular DNA damage was detected by comet assay^50^.

### In vitro cytotoxicity assessment

4T1 and HeLa cells were seeded into in 96-well cell culture plates at the density of 1 × 10^4^ cells/well, and incubated with various concentrations of nanoplates for 12 h at 37 °C in a humidified 5% CO_2_ atmosphere. Cells were illustrated with 0.2 or 0.5 W/cm^2^ 808 nm laser for 10 min. After incubation for another 24 h, the standard CCK-8 assay was executed to determine cell viability by collecting the absorbance at 450 nm on a microplate reader (BioTek). For proof of concept, UV (405 nm) irradiation was used instead of NIR irradiation for photocatalytic generation of holes and hydrogen molecules. The cytotoxicities of two sacrificial agents, AA and Na_2_S_4_O_6_, at different concentrations (31.2‒4000 µM) were evaluated, and 250 µM of concentration without visible cytotoxicity demonstration (Supplementary Fig. 9a) was chose for individual hole or hydrogen therapy (Supplementary Fig. 9b).

### In vivo tumor-targeted delivery and biodistribution measurement

All the in vivo experiments followed the protocols which were approved by the Animal Care and Use Committee of Shenzhen University. The 4T1 tumor-bearing mice model was established by injecting 10^7^ 4T1 cells into the hind limb of each female BALB/c mouse (about 20 g, purchased from Guangdong Medical Laboratory Animal Center). After tumors reached about 100 mm^3^, 100 μL PBS solution (10 mg/kg) of nanoplates was intravenously injected into 4T1 tumor-bearing mice. Main organs (heart, liver, spleen, lung, kidney, and blood) and tumors were extracted at fixed time points (1, 2, 4, 12, and 24 h) post injection. These organs were weighted and then completely digested with aqua regia, heated to dryness and finally diluted with deionized water. The quantitative analysis of Sn was determined by ICP-AES to measure the biodistribution of nanoplates (*n* = 3).

### In vivo tumor therapy assessment

For 4T1 tumor therapy, when tumor size reached approximately 80 mm^3^ (designed as day 0), the treatment was performed. Two experienced researchers randomly divided mice into six groups (*n* = 5), which were intravenously injected with 100 μL PBS containing or excluding SnS_2_/SnS_1.68_–WO_2.41_ nanoplates (10 mg/kg) followed with/without 808 nm laser irradiation (0.5 W/cm^2^, 20 min). 20 min 808 nm laser irradiation upon tumors was executed at day 1 and day 3. Body weight and tumor size of each mouse were recorded every other day. After 22 days of treatment, the tumors in the blank control group reached about 2000 mm^3^, and therefore all the mice were humanely executed. Main organs (heart, liver, spleen, lung, and kidney) and tumors were extracted for histological analysis by the hematoxylin and eosin (H&E) staining method. For HeLa tumor therapy, the HeLa tumor-bearing mice model was established by injecting 1 × 10^7^ HeLa cells into the hind limb of each female BALB/c nude mouse (about 15 g, purchased from Guangdong Medical Laboratory Animal Center). When tumor size reached approximate 60 mm^3^ (designed as day 0), the treatment was performed, which was similar to the case of 4T1 tumor therapy.

### Assessment of liver/kidney functions and hemotoxicity

Health BALB/c mice were randomly divided into 3 groups (*n* = 7) to be intravenously injected with 100 μL PBS (as control), 100 μL 100 mg/kg SnS_2_ nanoplates, and 100 μL 100 mg/kg SnS_1.68_-WO_2.41_ nanoplates, respectively. After 2 weeks, the blood was collected and assessed using a biochemical analyzer (iMagic-M7) and a blood cell analyzer (BC-31s, Mindray).

### Reporting summary

Further information on research design is available in the Nature Research Reporting Summary linked to this article.

## Supplementary information

Supplementary Information

Reporting Summary

## Data Availability

All the data supporting the findings of this study are available within the article and its Supplementary information files and from the corresponding author upon reasonable request.
