# Supplementary material for: Photocatalysis-mediated drug-free sustainable cancer therapy using nanocatalyst
Source: Nat Commun. 2021 Mar 1;12:1345. doi: 10.1038/s41467-021-21618-1 (PMC7921091; doi:10.1038/s41467-021-21618-1)
Supplement: Supplementary file 1 — Supplementary Information [file 41467_2021_21618_MOESM1_ESM.pdf]

Supplementary Information

Photocatalysis-mediated drug-free sustainable cancer therapy using nanocatalyst

Bin Zhao,[1,†] Yingshuai Wang,[1,†] Xianxian Yao,[1] Danyang Chen,[1,2] Mingjian Fan,[1] Zhaokui Jin,[1] Qianjun He[1,2]*

[1] Guangdong Provincial Key Laboratory of Biomedical Measurements and Ultrasound Imaging, National-Regional Key Technology Engineering Laboratory for Medical Ultrasound, Marshall Laboratory of Biomedical Engineering, School of Biomedical Engineering, Health Science Center, Shenzhen University, No. 1066 Xueyuan Road, Shenzhen 518060, Guangdong, China

[2] Center of Hydrogen Science, Shanghai Jiao Tong University, Shanghai 200240, China

*Corresponding author. Email: nanoflower@126.com

† These authors contributed equally to this work.

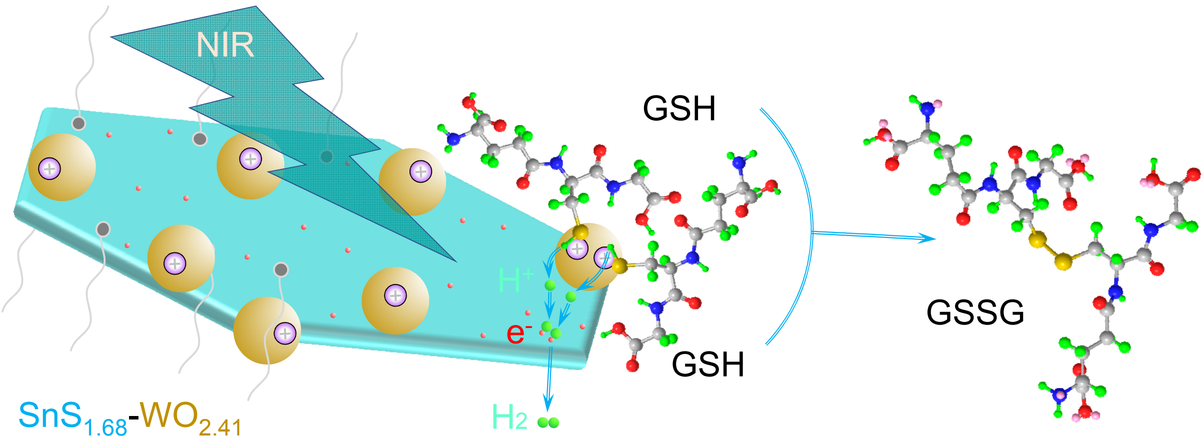

$$SnS_{1.68}\text{-}WO_{2.41} + h\nu \rightarrow e^-\text{-}SnS_{1.68}\text{-}WO_{2.41}\text{-}h^+$$

$$2GSH + 2h^+ \rightarrow GS\text{-}SG + 2H^+ \quad ; \quad 2H^+ + 2e^- \rightarrow H_2$$

$$\Rightarrow 2GSH \xrightarrow{SnS_{1.68}\text{-}WO_{2.41} + h\nu} GSSG + H_2$$

Supplementary Figure 1. Schematic illustration of NIR-photocatalytic GSH oxidation and hydrogen generation by the $SnS_{1.68}$-$WO_{2.41}$ nanocatalyst.

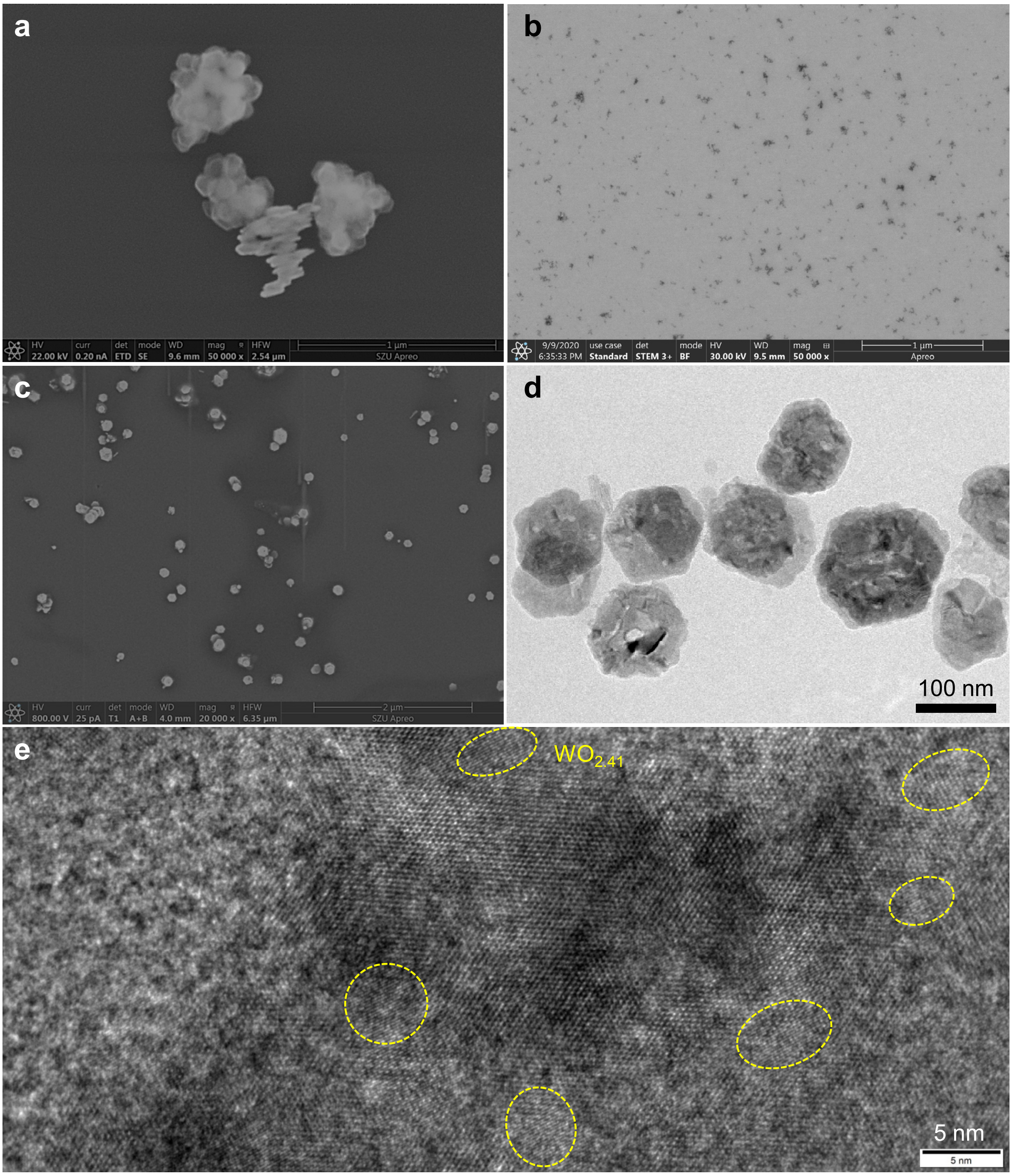

Supplementary Figure 2. SEM images of SnS_2 (**a**), $WO_{2.41}$ (**b**), and $SnS_{1.68}$-$WO_{2.41}$ (**c**) nanoplates, and TEM (**d**) and HR-TEM (**e**) images of $SnS_{1.68}$-$WO_{2.41}$ nanoplates. In the figure e, yellow dashed cycles point out $WO_{2.41}$ nanodots dispersed on $SnS_{1.68}$ nanoplates. The experiment was repeated three times independently with similar results.

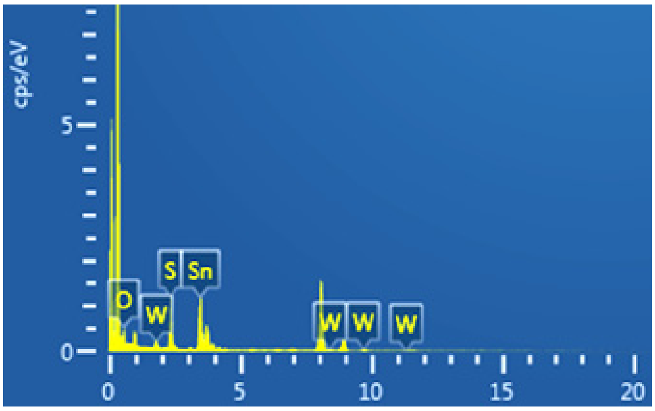

Supplementary Figure 3. EDX pattern of $SnS_{1.68}$-$WO_{2.41}$ nanoplates.

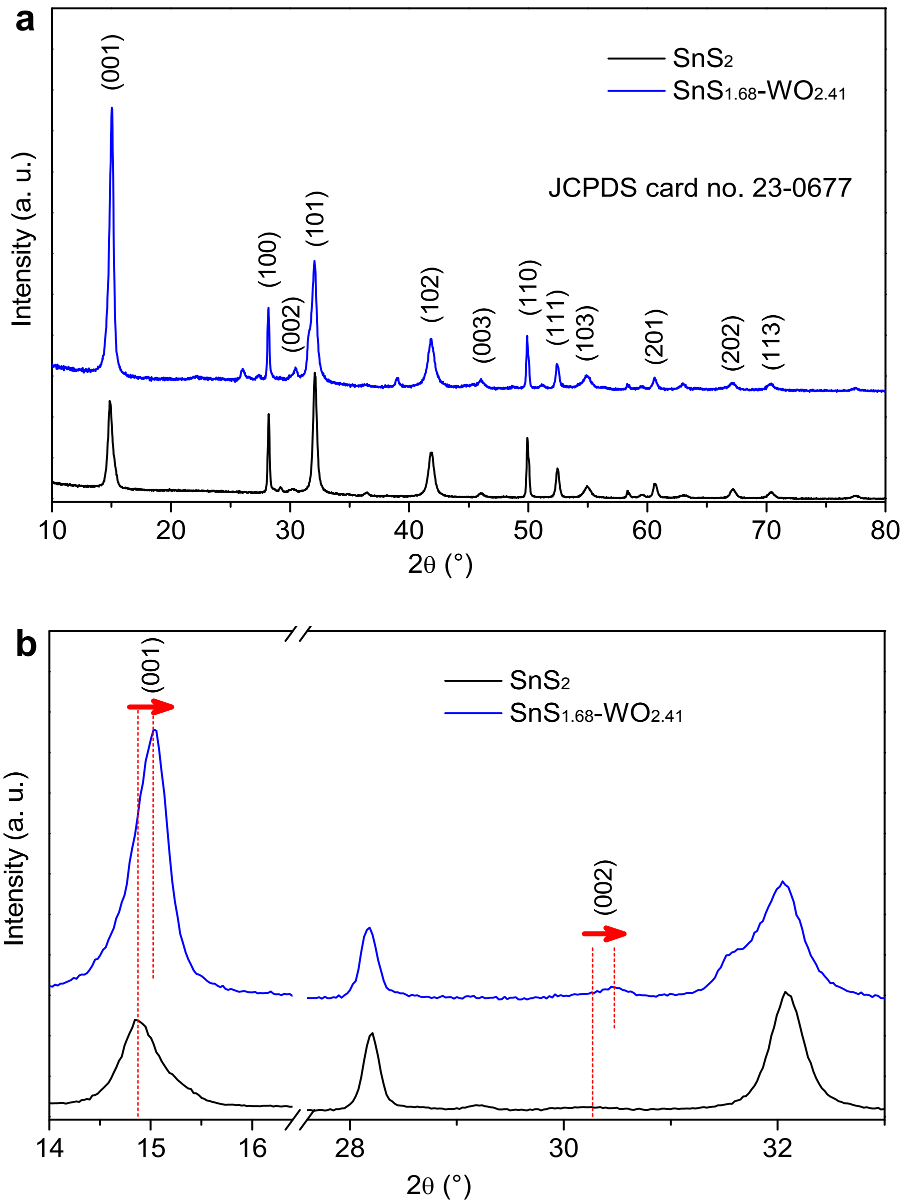

Supplementary Figure 4. XRD patterns of SnS_2 and $SnS_{1.68}$-$WO_{2.41}$ nanoplates (**a**), and magnified ones to demonstrate the shift of crystal lattice (**b**). As shown by red arrows in

figure b, from SnS_2 to $SnS_{1.68}$-$WO_{2.41}$, the shift of (001) and (002) planes to high angle means the slight change in the crystal lattice, reflecting the transition process of sulfur deficiency.

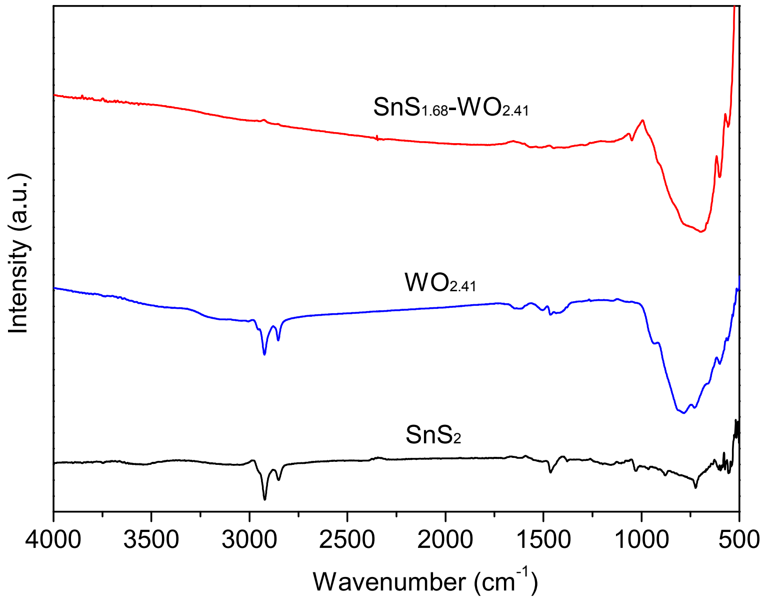

Supplementary Figure 5. FTIR spectra of SnS_2, $WO_{2.41}$ and $SnS_{1.68}$-$WO_{2.41}$.

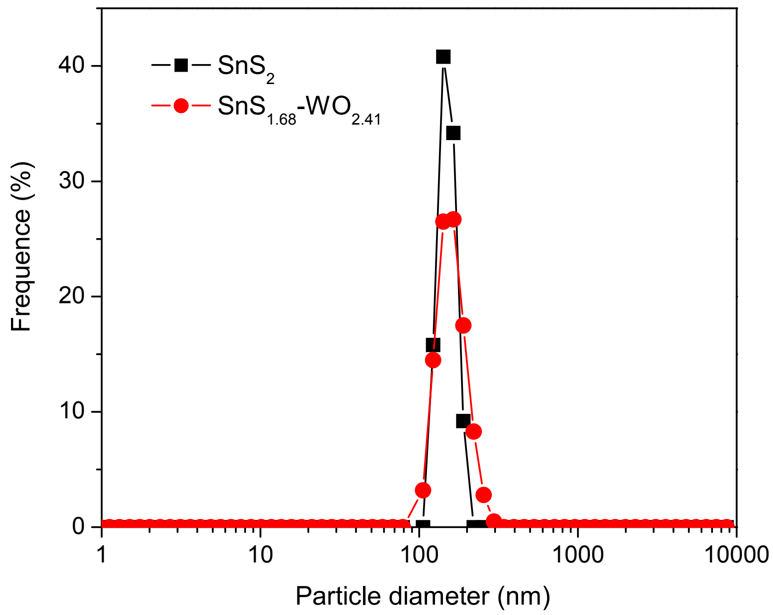

Supplementary Figure 6. DLS patterns of the aqueous solutions of PTMP-PMAAmodified SnS_2 and $SnS_{1.68}$-$WO_{2.41}$ nanoplates.

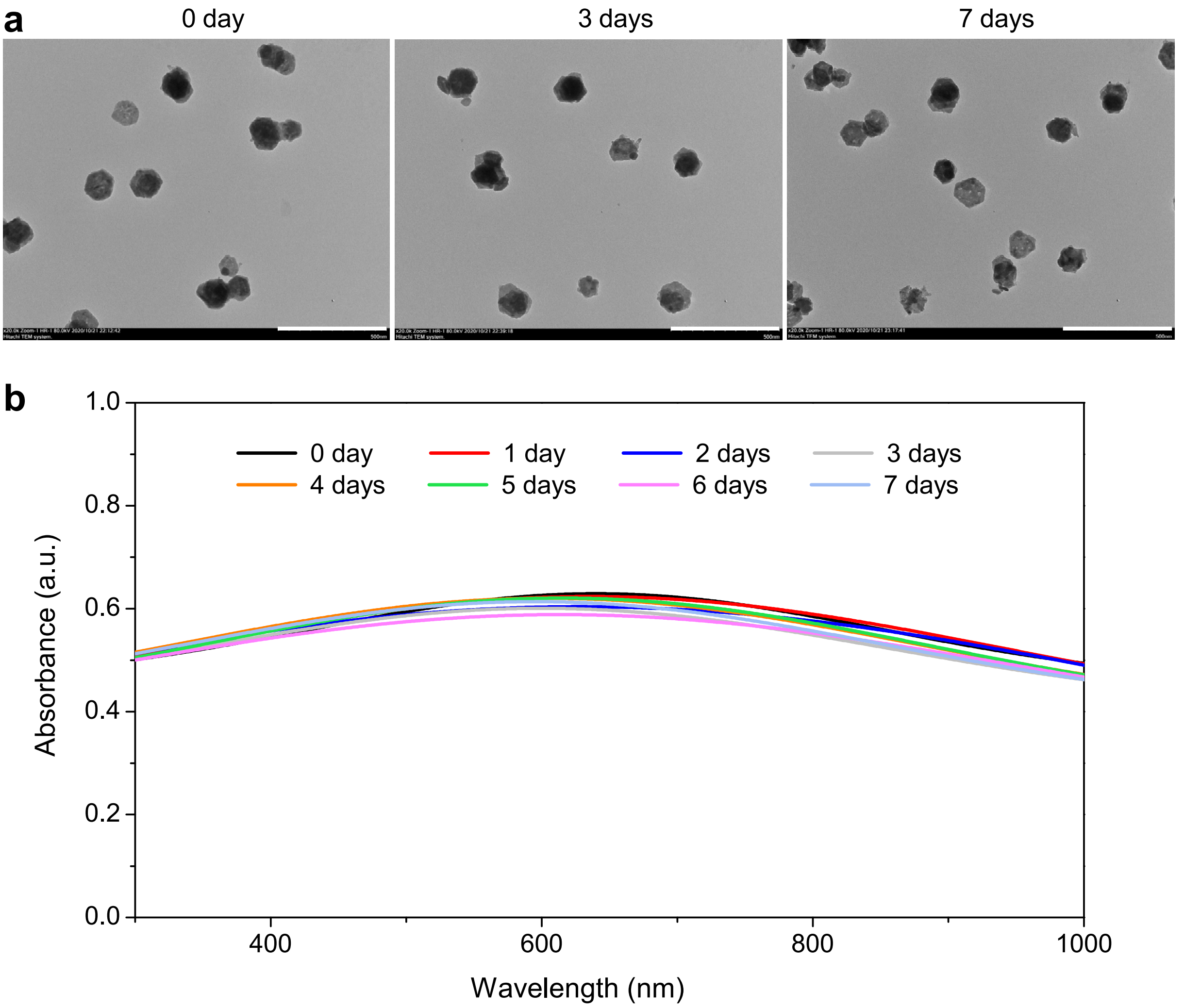

Supplementary Figure 7. The stability of $SnS_{1.68}$-$WO_{2.41}$ nanoplates in PBS: TEM images (**a**) and UV patterns (**b**) after immersion for different time periods. Scale bars, 500 nm. The experiment was repeated three times independently with similar results.

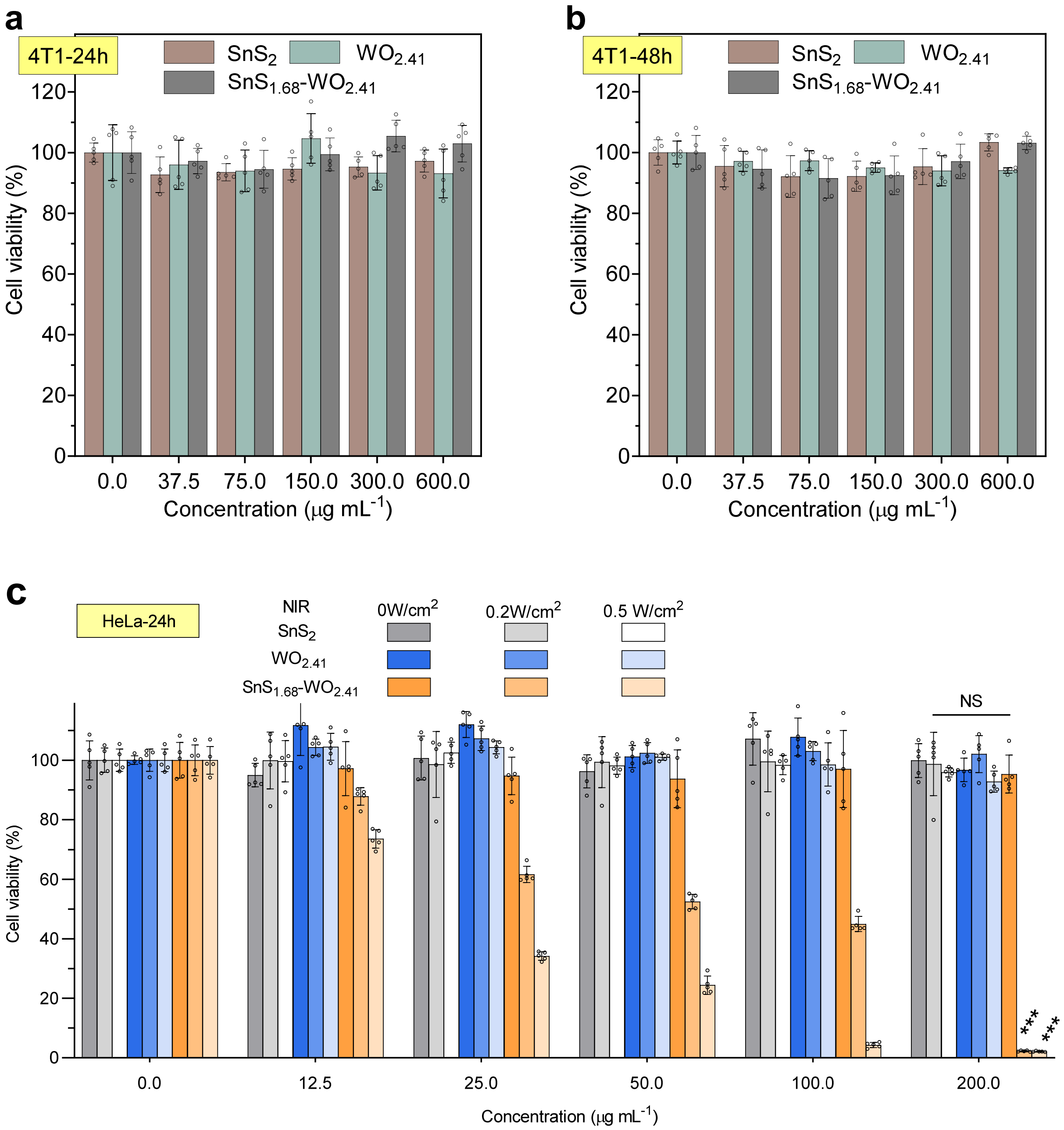

Supplementary Figure 8. The cytotoxicity of SnS_2, $WO_{2.41}$ and $SnS_{1.68}$-$WO_{2.41}$ nanoplates to 4T1 cells (**a**: 24 h incubation (*n*=5 biologically independent samples); **b**: 48 h incubation (*n*=5 biologically independent samples)) and HeLa cells (**c**: 24 h incubation (*n*=5 biologically independent samples)). *P* values were calculated by the two-tailed Student's *t*-test (***P<0.000001; NS, no significant difference). Data are presented as mean values ± SD. Source data are provided as a Source Data file.

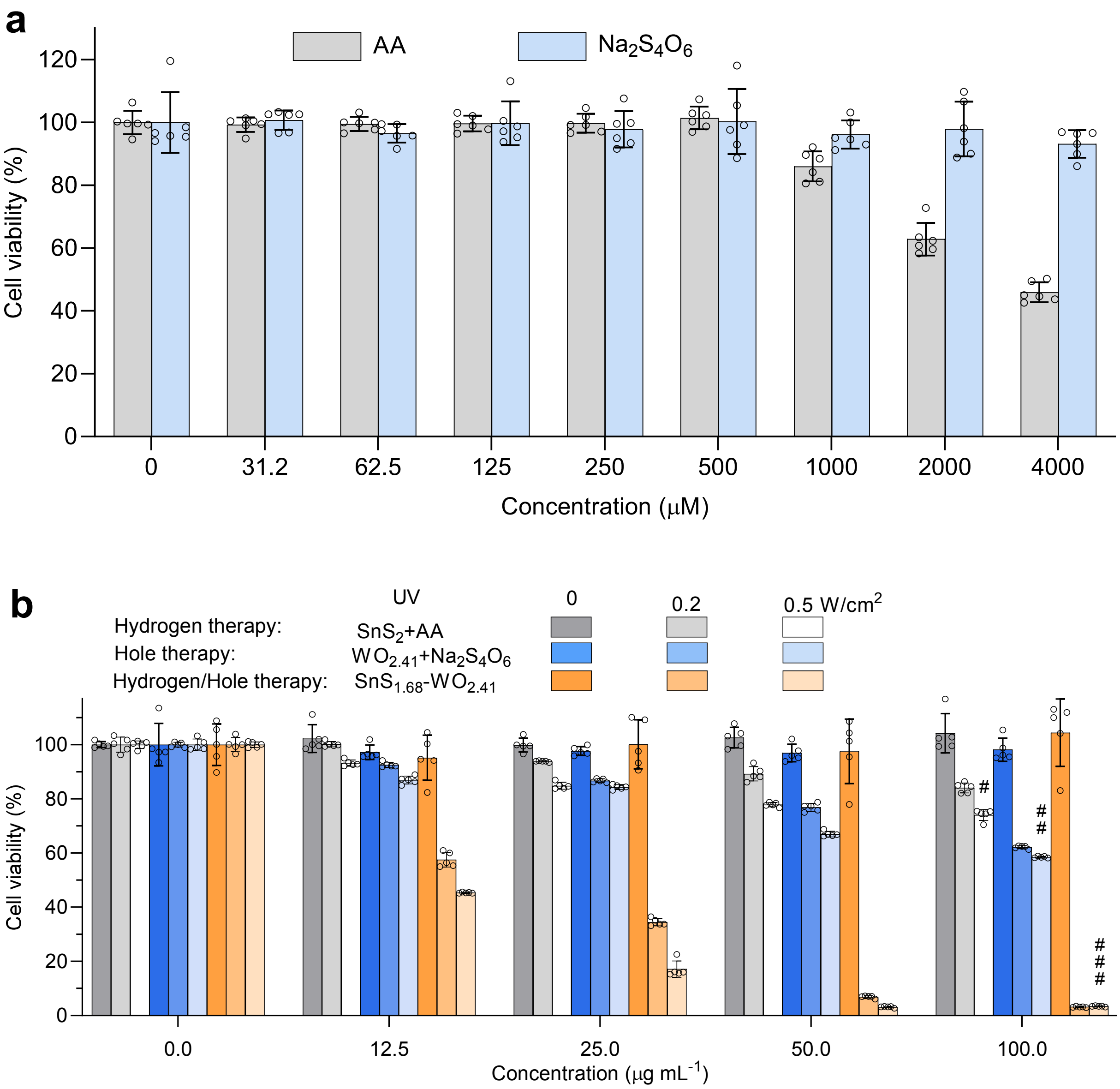

Supplementary Figure 9. The cytotoxicities of sacrificial agents AA and $Na_2S_4O_6$ (n=6 biologically independent samples) (**a**), and SnS_2 and $WO_{2.41}$ nanoparticles to 4T1 cells in the absence and presence of UV irradiation (n=5 biologically independent samples) (**b**). P values were calculated by the two-tailed Student's t-test (#P=0.000081, ##P=0.000002, ###P<0.000001). Data are presented as mean values ± SD. Source data are provided as a Source Data file.

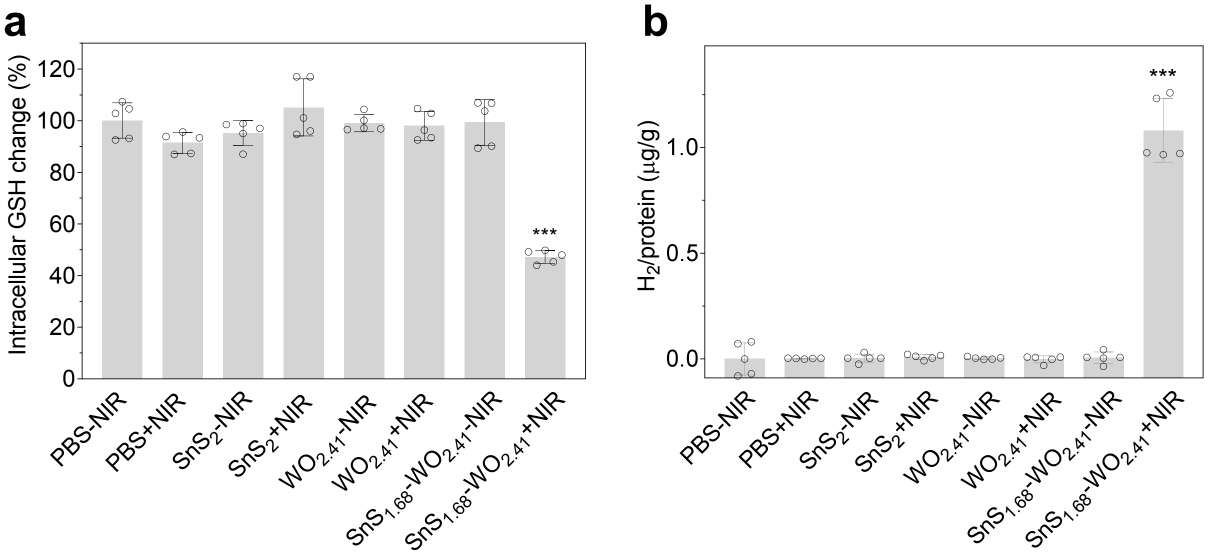

Supplementary Figure 10. The changes in intracellular levels of GSH (*n*=5 biologically independent samples) (**a**) and hydrogen molecule (**b**) in nanoplates-treated 4T1 cells before and after NIR irradiation (*n*=5 biologically independent samples). *P* values were calculated by the two-tailed Student's *t*-test (***$P<0.000001$). Data are presented as mean values ± SD. Source data are provided as a Source Data file.

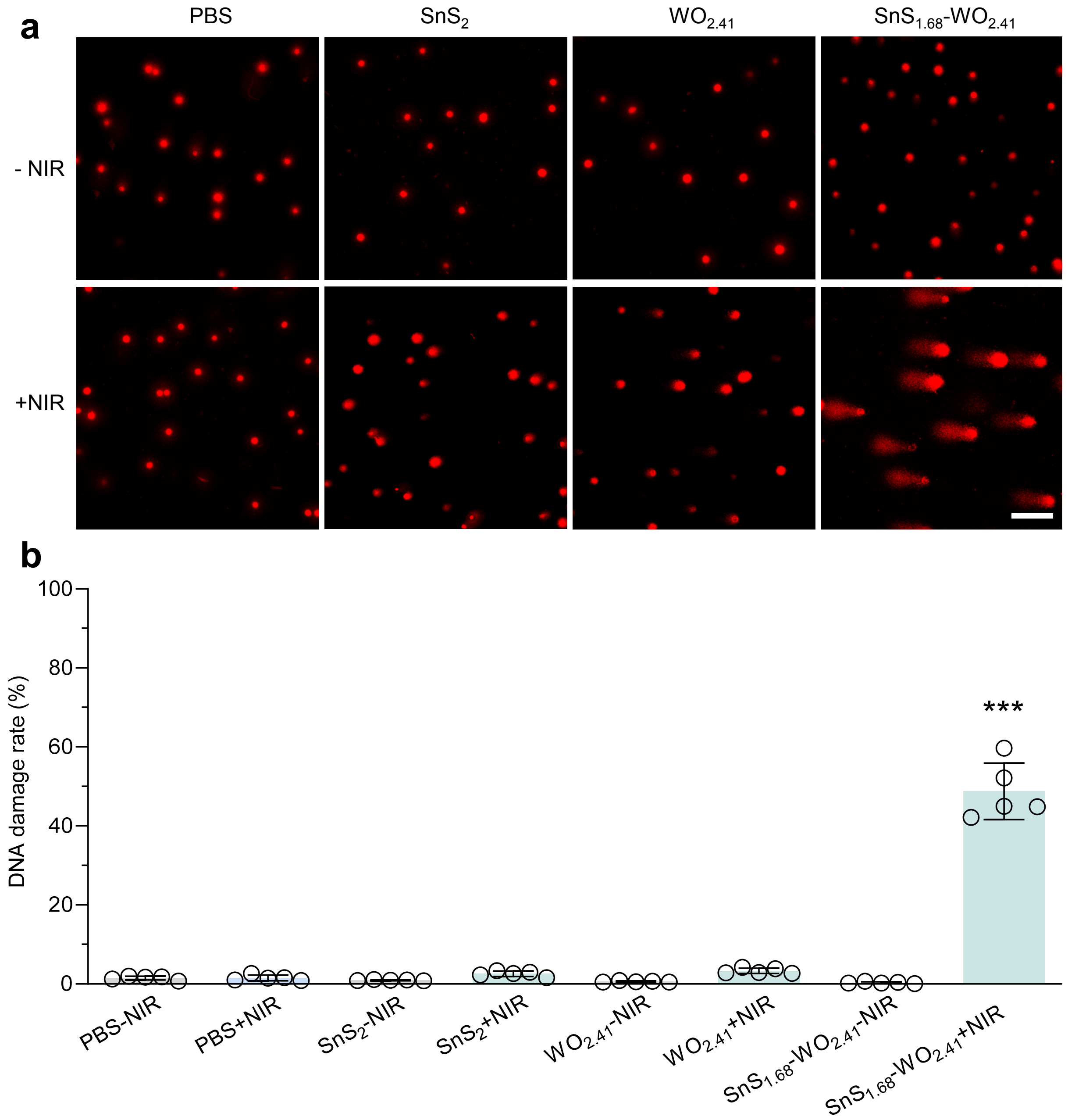

Supplementary Figure 11. Intracellular DNA damage of 4T1 cells after different treatments which was detected by comet assay (**a**), and the qualification analysis (n=5 biologically independent samples) (**b**). P values were calculated by the two-tailed Student's t-test (***P<0.000001). Scale bar, 100 μm. Data are presented as mean values ± SD. Source data are provided as a Source Data file.

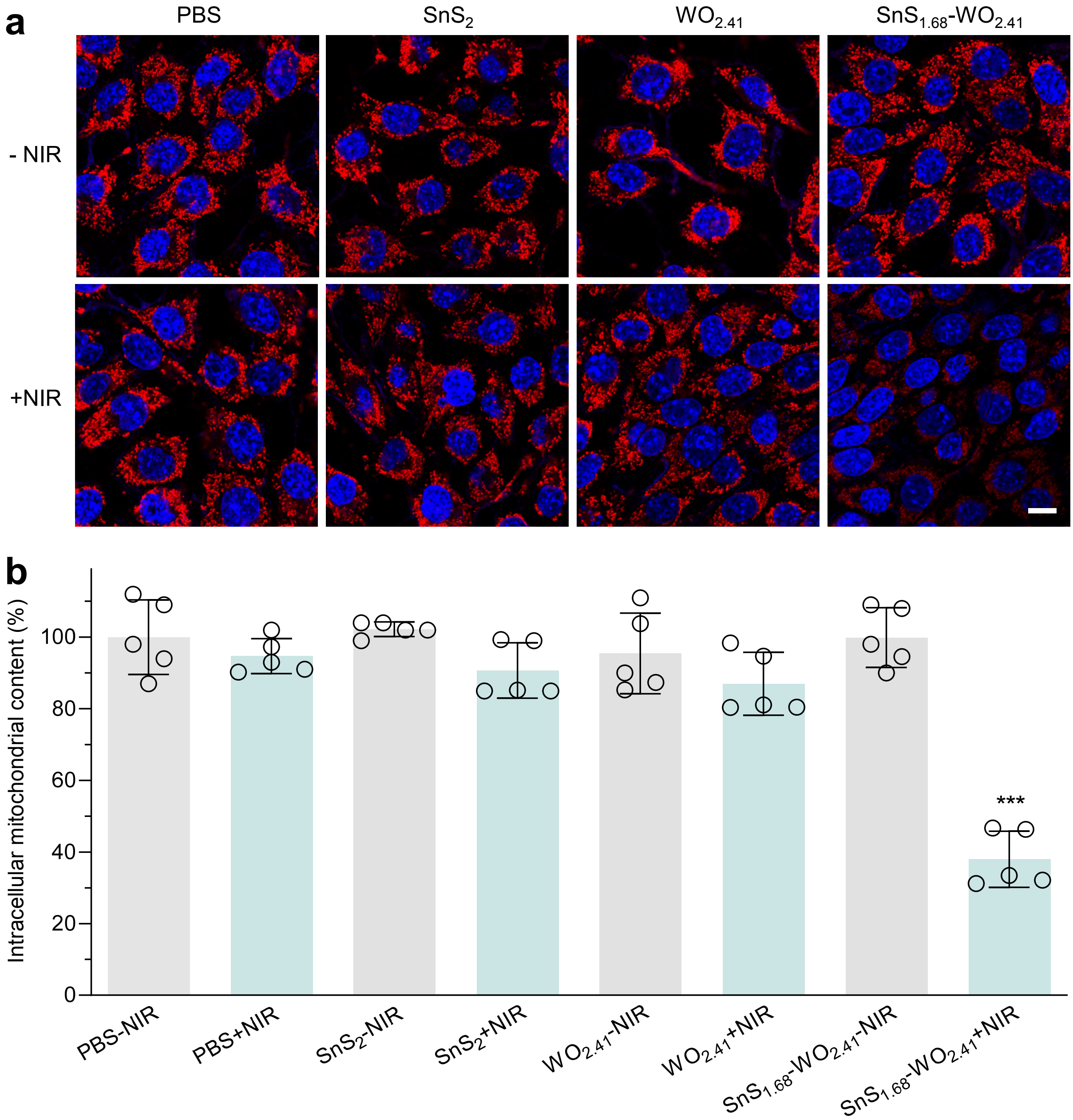

Supplementary Figure 12. Confocal images of treated 4T1 cells with mitochondria and nuclei dyed with red and blue (**a**, Scale bar, 10 μm), and the intracellular mitochondrial contents in 4T1 cells after different treatments, which were calculated from confocal images by ImageJ software (n=5 biologically independent samples) (**b**). P values were calculated by the two-tailed Student's t-test (***P=0.000005). Data are presented as mean values ± SD. Source data are provided as a Source Data file.

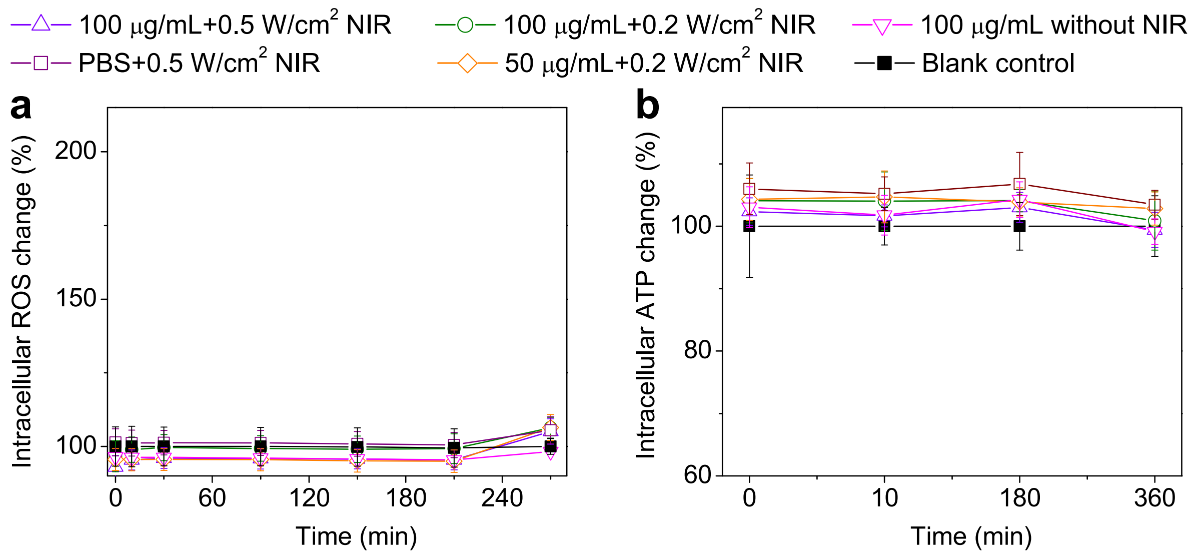

Supplementary Figure 13. The intracellular ROS (**a**) and ATP (**b**) monitoring (n=6 biologically independent samples) in SnS_2-treated 4T1 cells in the presence/absence of NIR irradiation (808 nm, 0.2 or 0.5 W/cm^2, 10 min). Data are presented as mean values ± SD. Source data are provided as a Source Data file.

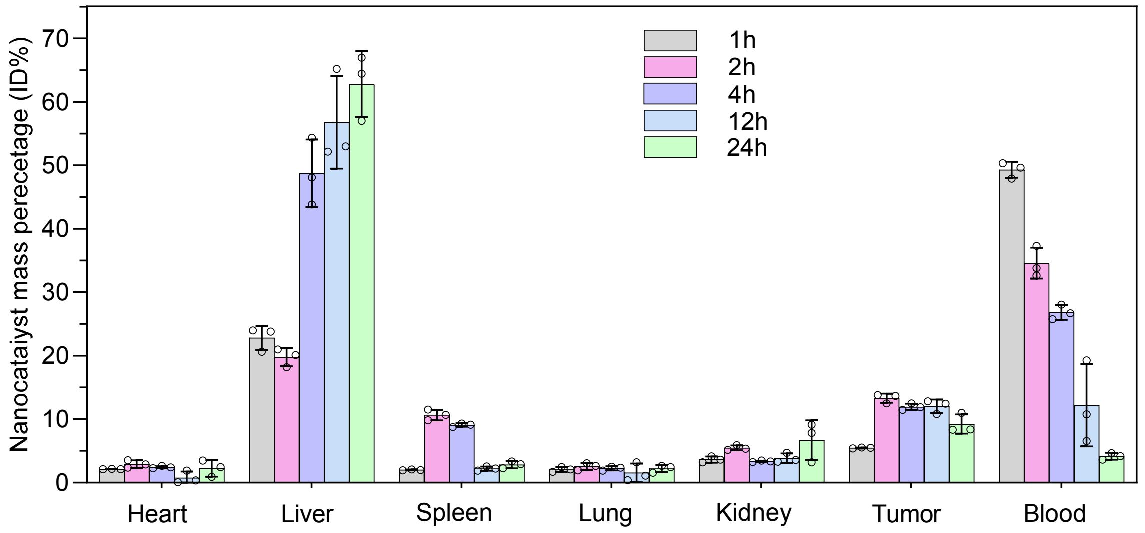

Supplementary Figure 14. The biodistribution of $SnS_{1.68}$-$WO_{2.41}$ nanocatalyst in 4T1 tumor-bearing mice after intravenous injection by ICP measurement (n=3 independent experiments). Data are presented as mean values ± SD. Source data are provided as a Source Data file.

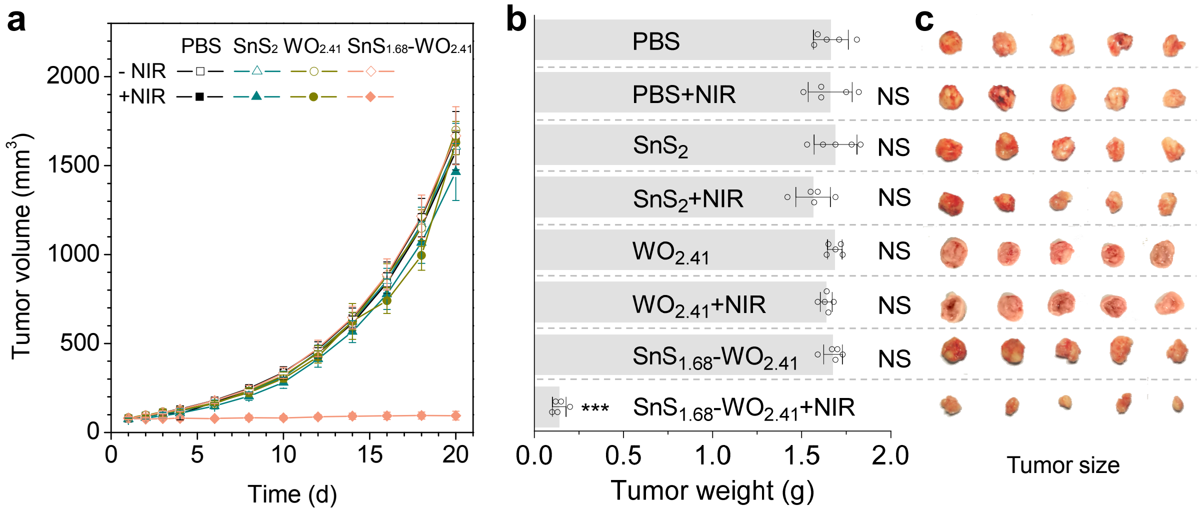

Supplementary Figure 15. The monitoring of HeLa tumor volume (n=5 biologically independent samples) (**a**), the weight (n=5 biologically independent samples) (**b**) and size (**c**) of extracted tumors after 20 day treatment. P values were calculated by the two-tailed Student's t-test (***P<0.000001; NS, no significant difference). Data are presented as mean values ± SD. Source data are provided as a Source Data file.

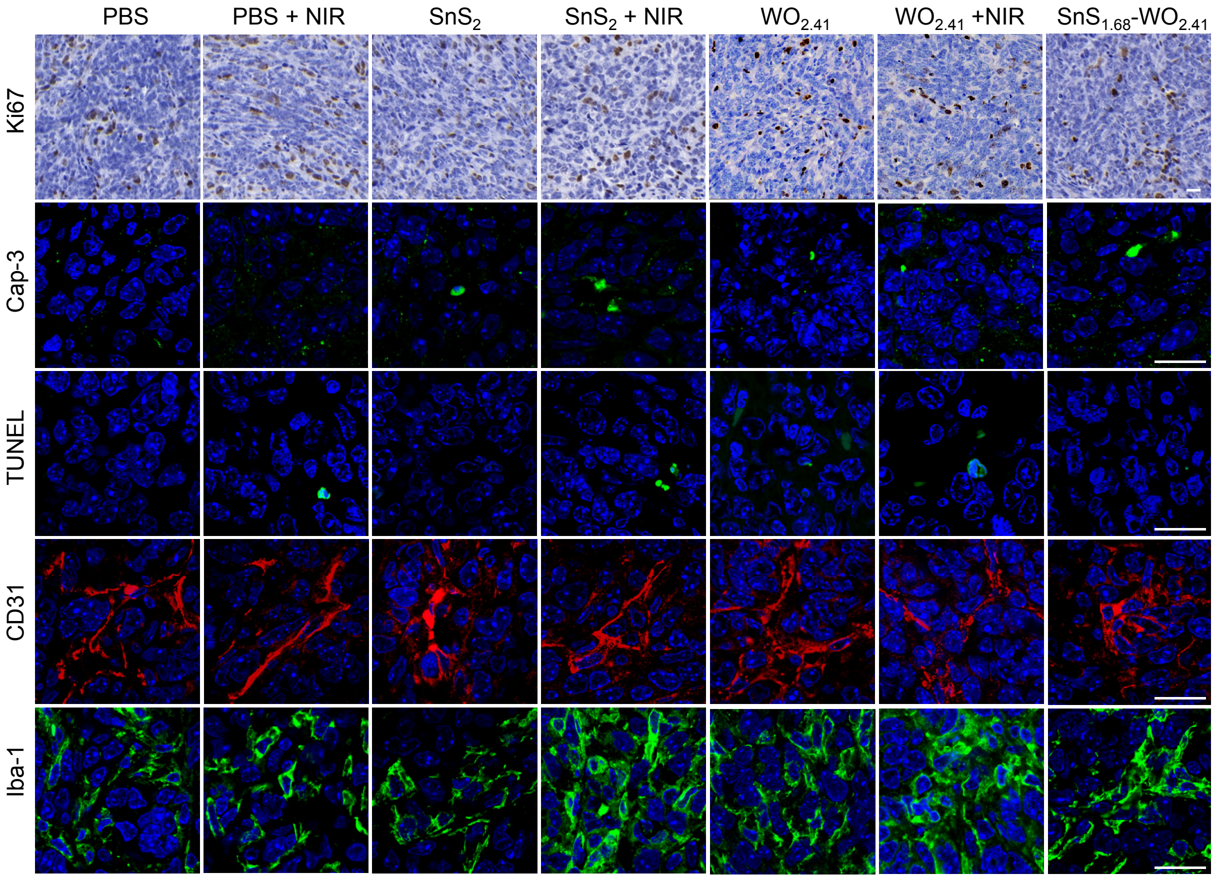

Supplementary Figure 16. Immunohistochemical analysis of Ki67$^+$ proliferating cells,

caspase-3^{+} apoptotic cells, TUNEL^{+} apoptotic cells, CD31^{+} tumor vessels, and IBA1^{+} tumor-associated macrophagesin in 4T1 tumors with different treatments. Scale bars, 20 µm. The experiment was repeated three times independently with similar results.

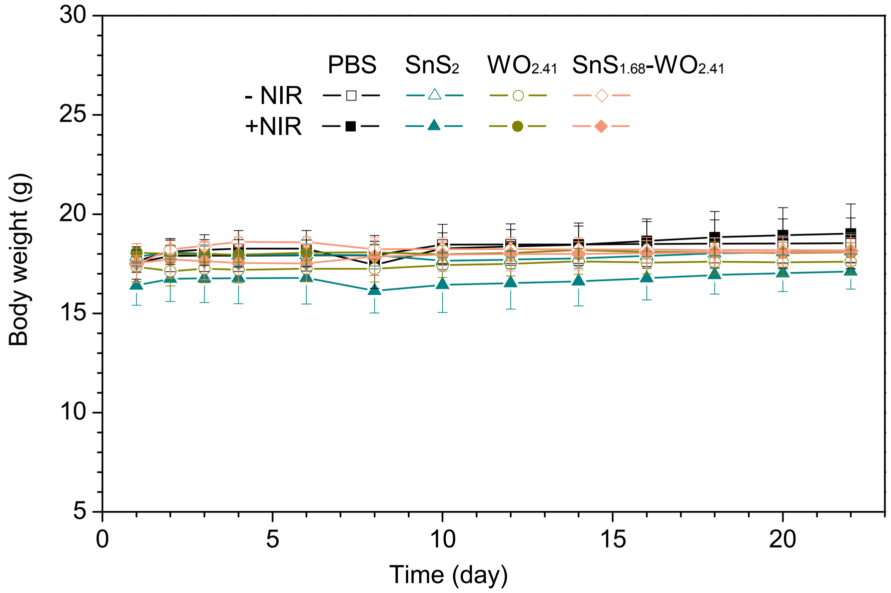

Supplementary Figure 17. Body weight change of 4T1 tumor-bearing mice during treatment (*n*=5 biologically independent samples). Data are presented as mean values ± SD. Source data are provided as a Source Data file.

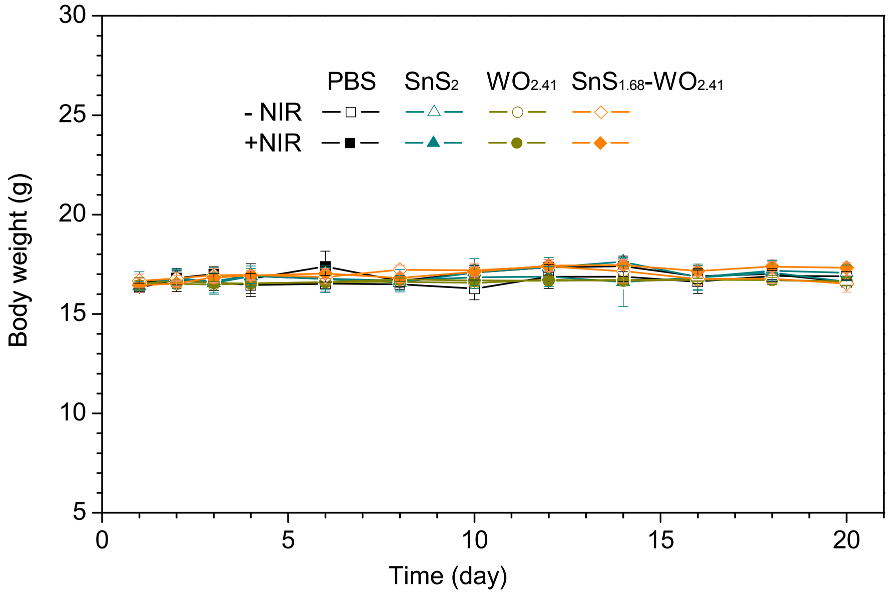

Supplementary Figure 18. Body weight change of HeLa tumor-bearing mice during treatment (*n*=5 biologically independent samples). Data are presented as mean values ± SD. Source data are provided as a Source Data file.

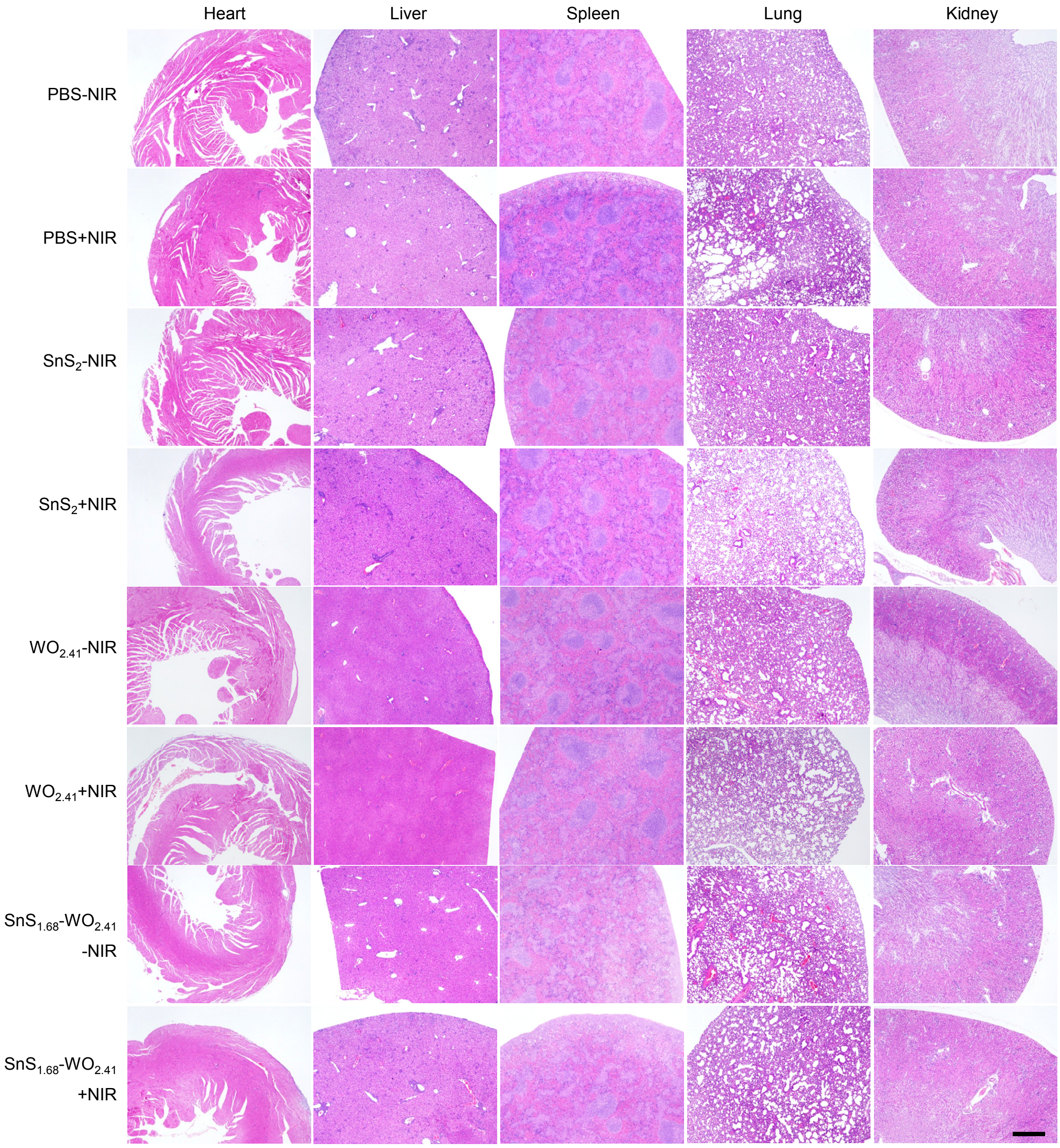

Supplementary Figure 19. Histological examination of main organs (heart, liver, spleen, lung and kidney) from 4T1 tumor-bearing mice after different treatments by the HE staining method. Scale bar, 500 μm. The experiment was repeated three times independently with similar results.

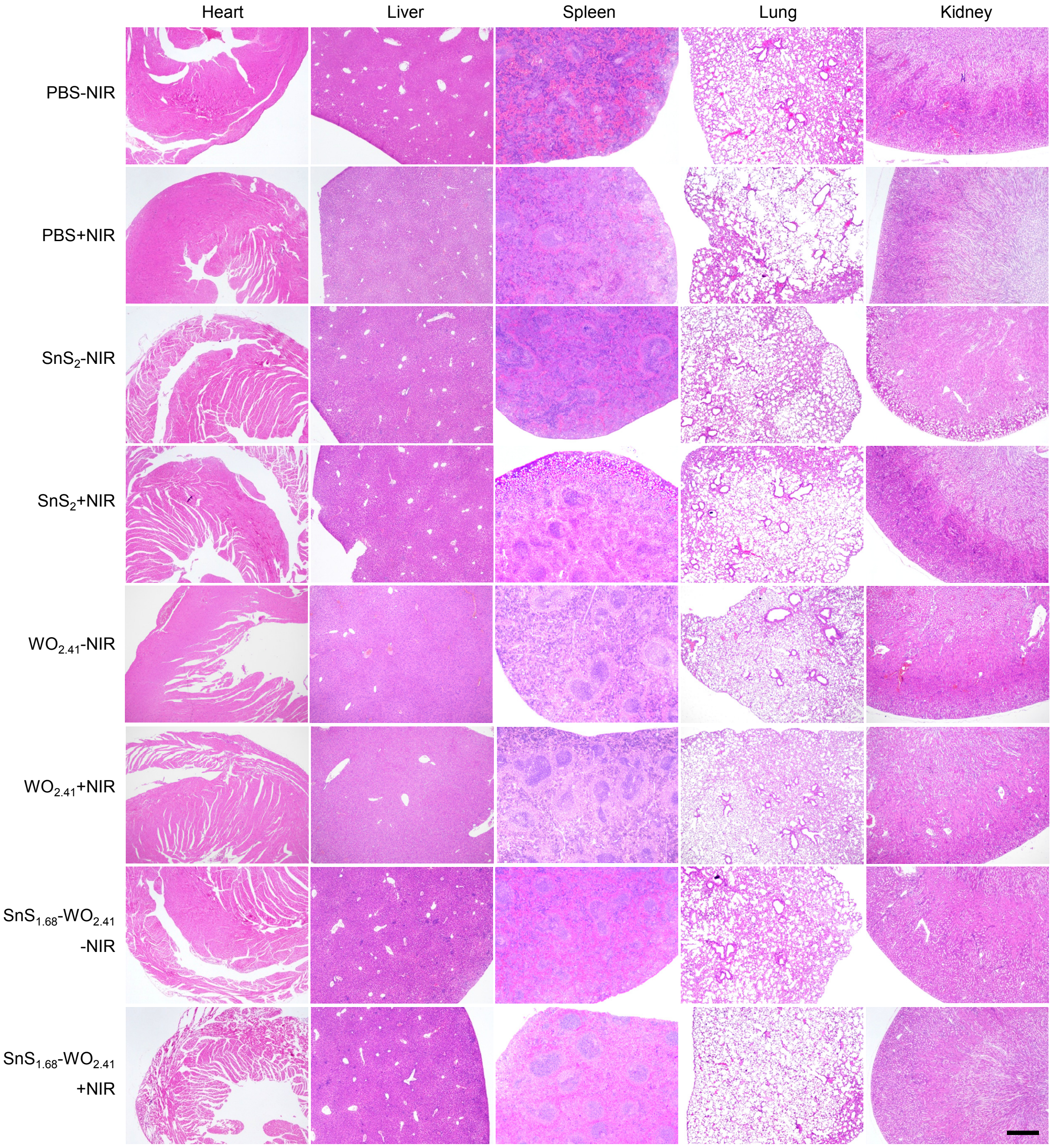

Supplementary Figure 20. Histological examination of main organs (heart, liver, spleen, lung and kidney) from HeLa tumor-bearing mice after different treatments by the HE staining method. Scale bar, 500 µm. The experiment was repeated three times independently with similar results.

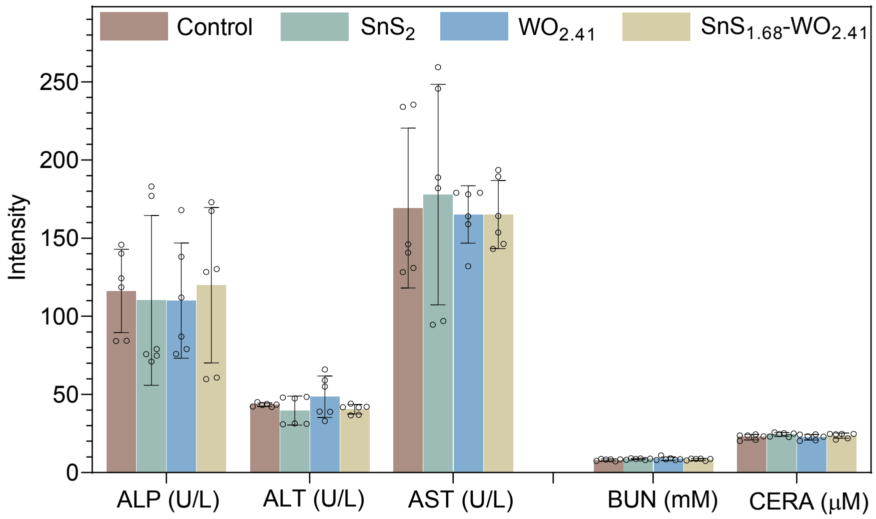

Supplementary Figure 21. Blood biochemical analyses of liver/kidney functions. ALP, alkaline phosphatase; ALT, alanine transaminase; AST, aspartate transaminase; CREA, creatinine; BUN, blood urea nitrogen (n=6 biologically independent samples). Mean value and error bar are defined as mean and s.d., respectively.

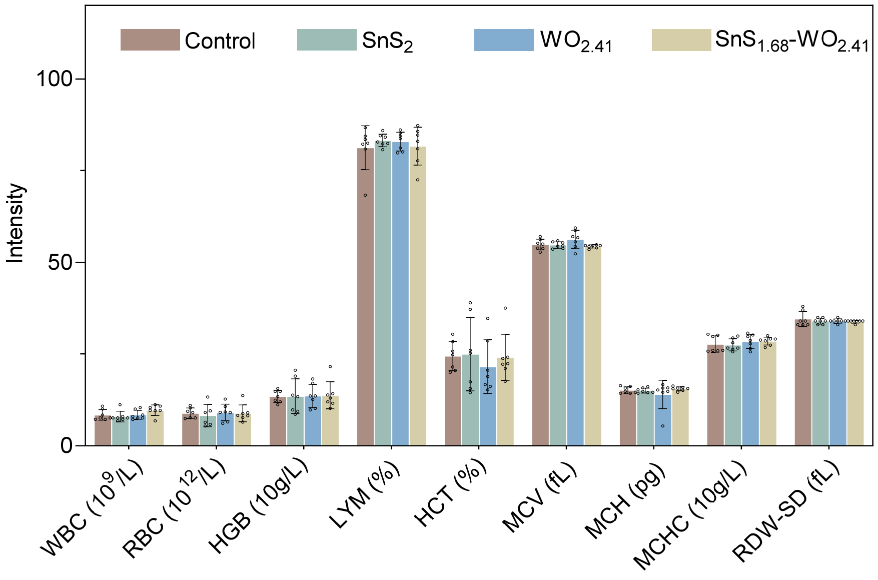

Supplementary Figure 22. The assessment of standard haematology markers including white blood cells (WBC), red blood cells (RBC), hemoglobin (HGB), hematocrit (HCT), mean corpuscular volume (MCV), mean corpuscular hemoglobin (MCH), mean corpuscular hemoglobin concentration (MCHC), red blood cell volume distribution width (RDW-SD) and lymphocytes percentage (LYM) (n=7 biologically independent samples). Mean value and error bar are defined as mean and s.d., respectively.
