# Supplementary material for: Photocatalysis-mediated drug-free sustainable cancer therapy using nanocatalyst
Source: Nat Commun. 2021 Mar 1;12:1345. doi: 10.1038/s41467-021-21618-1 (PMC7921091; doi:10.1038/s41467-021-21618-1)
Supplement: Supplementary file 2 — Reporting Summary [file 41467_2021_21618_MOESM2_ESM.pdf]

nature research

Corresponding author(s): Qianjun He
NCOMMS-20-21953A

Last updated by author(s): Jan 5, 2021

Reporting Summary

Nature Research wishes to improve the reproducibility of the work that we publish. This form provides structure for consistency and transparency in reporting. For further information on Nature Research policies, see our Editorial Policies and the Editorial Policy Checklist.

Statistics

For all statistical analyses, confirm that the following items are present in the figure legend, table legend, main text, or Methods section.

n/a	Confirmed	
☐	☒	The exact sample size (*n*) for each experimental group/condition, given as a discrete number and unit of measurement
☐	☒	A statement on whether measurements were taken from distinct samples or whether the same sample was measured repeatedly
☐	☒	The statistical test(s) used AND whether they are one- or two-sided<br>*Only common tests should be described solely by name; describe more complex techniques in the Methods section.*
☒	☐	A description of all covariates tested
☒	☐	A description of any assumptions or corrections, such as tests of normality and adjustment for multiple comparisons
☐	☒	A full description of the statistical parameters including central tendency (e.g. means) or other basic estimates (e.g. regression coefficient) AND variation (e.g. standard deviation) or associated estimates of uncertainty (e.g. confidence intervals)
☐	☒	For null hypothesis testing, the test statistic (e.g. *F*, *t*, *r*) with confidence intervals, effect sizes, degrees of freedom and *P* value noted<br>*Give P values as exact values whenever suitable.*
☒	☐	For Bayesian analysis, information on the choice of priors and Markov chain Monte Carlo settings
☒	☐	For hierarchical and complex designs, identification of the appropriate level for tests and full reporting of outcomes
☒	☐	Estimates of effect sizes (e.g. Cohen's *d*, Pearson's *r*), indicating how they were calculated

Our web collection on statistics for biologists contains articles on many of the points above.

Software and code

Policy information about availability of computer code

Data collection	TXT
Data analysis	Origin 8.5, GraphPad Prism 9, Microsoft Office PowerPoint 2007

For manuscripts utilizing custom algorithms or software that are central to the research but not yet described in published literature, software must be made available to editors and reviewers. We strongly encourage code deposition in a community repository (e.g. GitHub). See the Nature Research guidelines for submitting code & software for further information.

Data

Policy information about availability of data

All manuscripts must include a data availability statement. This statement should provide the following information, where applicable:
- Accession codes, unique identifiers, or web links for publicly available datasets
- A list of figures that have associated raw data
- A description of any restrictions on data availability

All data needed to evaluate the conclusions in the paper are present in the paper and/or the Supplementary Materials. Additional data related to this paper may be requested from the authors.

Field-specific reporting

Life sciences study design

All studies must disclose on these points even when the disclosure is negative.

Sample size	The sample size (generally n≥5 biologically independent samples) was determined by allowable error size and accuracy, and resources.
Data exclusions	No data were excluded from the analyses.
Replication	The experimental findings were repeatable with independent samples.
Randomization	The samples were randomly grouped.
Blinding	The investigators were blinded to group allocation during data collection and analysis.

Reporting for specific materials, systems and methods

We require information from authors about some types of materials, experimental systems and methods used in many studies. Here, indicate whether each material, system or method listed is relevant to your study. If you are not sure if a list item applies to your research, read the appropriate section before selecting a response.

Materials & experimental systems

n/a	Involved in the study	
☒	☐	Antibodies
☐	☒	Eukaryotic cell lines
☒	☐	Palaeontology and archaeology
☐	☒	Animals and other organisms
☒	☐	Human research participants
☒	☐	Clinical data
☒	☐	Dual use research of concern

Methods

n/a	Involved in the study	
☒	☐	ChIP-seq
☒	☐	Flow cytometry
☒	☐	MRI-based neuroimaging

Eukaryotic cell lines

Policy information about cell lines

Cell line source(s)	The mouse 4T1 breast cancer cells and human HeLa cervical carcinoma cells were purchased from China Type Culture Collection (CTCC) obtained from the American Type Culture Collection (ATCC).
Authentication	The use of 4T1 and HeLa cells was authenticated by China Type Culture Collection (CTCC).
Mycoplasma contamination	All the cell lines routinely tested negative for mycoplasma contamination using MycoSET Mycoplasma real-time PCR detection Kit.
Commonly misidentified lines (See ICLAC register)	No commonly misidentified cell lines were used in the study.

Animals and other organisms

Policy information about studies involving animals; ARRIVE guidelines recommended for reporting animal research

Laboratory animals	All healthy female BALB/c mice (4 weeks old) were purchased from Guangdong Medical Laboratory Animal Center. All mice were kept in specific pathogen-free (SPF) conditions where they were maintained at 22°C and on a 12-hour light-dark cycle (7am to 7pm) and given food and water adlibitum.
Wild animals	The study did not involve wild animals.
Field-collected samples	The study did not involve any samples collected from field.
Ethics oversight	All the in vivo experiments followed the protocols which were approved by the Animal Care and Use Committee of Shenzhen University.

Note that full information on the approval of the study protocol must also be provided in the manuscript.
